# The pitfalls of biodiversity proxies: Differences in richness patterns of birds, trees and understudied diversity across Amazonia

**DOI:** 10.1038/s41598-019-55490-3

**Published:** 2019-12-16

**Authors:** Camila D. Ritter, Søren Faurby, Dominic J. Bennett, Luciano N. Naka, Hans ter Steege, Alexander Zizka, Quiterie Haenel, R. Henrik Nilsson, Alexandre Antonelli

**Affiliations:** 1 Department of Eukaryotic Microbiology, University of Duisburg-Essen, Universitätsstrasse 5 S05 R04 H83, D-45141 Essen, Germany; 2 Gothenburg Global Biodiversity Centre, Box 461, SE-405 30 Göteborg, Sweden; 3 Department of Biological and Environmental Sciences, University of Gothenburg, Box 463, SE-405 30 Göteborg, Sweden; 4 Laboratório de Ornitologia, Departamento de Zoologia, Universidade Federal de Pernambuco, Recife, PE Brazil; 5 Naturalis Biodiversity Center, Leiden, Netherlands; 6 Systems Ecology, Free University, Amsterdam, Netherlands; 7 German Centre for Integrative Biodiversity Research (iDiv) Halle-Jena-Leipzig, Deutscher Platz 5e, 04103 Leipzig, Germany; 8 Zoological Institute, University of Basel, Vesalgasse 1, CH-4051 Basel, Switzerland; 9 Royal Botanic Gardens, Kew, TW9 3AE Richmond, Surrey UK

**Keywords:** Forest ecology, Ecosystem ecology, Microbial ecology

## Abstract

Most knowledge on biodiversity derives from the study of charismatic macro-organisms, such as birds and trees. However, the diversity of micro-organisms constitutes the majority of all life forms on Earth. Here, we ask if the patterns of richness inferred for macro-organisms are similar for micro-organisms. For this, we barcoded samples of soil, litter and insects from four localities on a west-to-east transect across Amazonia. We quantified richness as Operational Taxonomic Units (OTUs) in those samples using three molecular markers. We then compared OTU richness with species richness of two relatively well-studied organism groups in Amazonia: trees and birds. We find that OTU richness shows a declining west-to-east diversity gradient that is in agreement with the species richness patterns documented here and previously for birds and trees. These results suggest that most taxonomic groups respond to the same overall diversity gradients at large spatial scales. However, our results show a different pattern of richness in relation to habitat types, suggesting that the idiosyncrasies of each taxonomic group and peculiarities of the local environment frequently override large-scale diversity gradients. Our findings caution against using the diversity distribution of one taxonomic group as an indication of patterns of richness across all groups.

## Introduction

Despite significant advances in our understanding of global biodiversity, a fundamental question remains poorly understood^1^: *Do the same ecological patterns apply to macro and micro-organisms?* In fact, our understanding of biodiversity is biased towards charismatic and relatively easily identifiable taxa. For instance, for birds and flowering plants, an estimated 98%^2,3^ and 69%^3^ respectively of the extant species have been formally described. Yet, even in these taxonomically well-described groups, the geographic distribution of many species remains poorly understood (the ‘Wallacean shortfall’^4^). The overwhelming majority of the extant biodiversity, however, does not belong to these groups. All vertebrates combined represent only 0.7%, and all flowering plants only 3%, of the total estimated number of eukaryotic species. Many species, particularly of invertebrates and micro-organisms, are yet to be described (the ‘Linnaean shortfall’^4^) and their distribution has yet to be documented.

A pre-requisite to overcoming these shortfalls is the ability to record and recognize species. Species identification, however, requires taxonomic expertise, which in turn requires a substantial and long-term investment of resources, time and infrastructure, especially when species are vouchered and deposited in natural history collections^5^. Recently, high-throughput DNA sequencing approaches, in combination with DNA metabarcoding^6^, have enabled the identification of organisms and the estimation of diversity from bulk (unsorted) biological samples, facilitating the documentation of spatial diversity patterns across the tree of life^7,8^.

Besides geographic differences, large-scale biodiversity patterns vary among taxonomic groups. Some studies have already assessed the correlations between the diversity of macro and micro-organisms. On a global scale, a mismatch of diversity was found between below-ground organisms (bacteria, fungi and mesofauna) and above-ground organisms (mammal, birds, amphibians and vascular plants)^9^. Furthermore, bacterial diversity was higher in temperate regions, while fungi showed a weak latitudinal pattern^10^. In another study, fungal diversity displayed a latitudinal gradient but was uncorrelated with plant diversity^11^. For Neotropical forests, protists showed the same pattern of diversity as macro-organisms^12^, and fungi and bacteria followed the elevational gradient of diversity in the Andes^13^. The pattern of richness of fungi and bacteria in the mineral soil, however, was different from that of plants, not linear, with fungi having the lowest richness in median elevation and bacteria the highest^13^. In this context, the congruence or divergence in diversity across taxa remains unclear. This is problematic, since micro-organims are the most diverse and abundant groups in any habitat^14^ and are essential for ecosystem function^15^ and the fitness of higher organisms^16^, meaning that general insights into the distribution and drivers of diversity require their inclusion^17^.

Although insufficient biological knowledge prevails in nearly all ecosystems around the world, this problem is most conspicuous in tropical environments, and in particular in tropical forests. Amazonia is the world’s largest and most biodiverse tropical forest. On a large spatial scale, most macroscopic taxa show consistent patterns of diversity, possibly as a response to abiotic conditions and processes^18–20^. In this region, one of the most conspicuous patterns of species richness in well-studied groups, such as birds and trees, is a west-to-east diversity gradient: from the highly diverse areas on the eastern Andean slopes to the relatively less diverse areas on the Guiana Shield in the north and eastern Amazonian lowlands^18–22^. Several explanations for this pattern have been suggested, including the effects of marine incursions^23–25^, bedrock geology^26^, mountain base formation^18^, soil fertility^18,27^ and, more recently, a diversification process driven by moisture^28^.

While most of Amazonia is covered by lowland non-flooded terra-firme forests, several other vegetation types, such as flooded forests or white sand ecosystems, are common and widespread throughout the basin. Patterns of plant and avian diversity vary dramatically with vegetation type; as a general rule, terra-firme forests are more diverse than seasonally flooded forests^29–31^. Forests that are seasonally flooded by nutrient-rich, white-water rivers (várzeas) are more diverse than forests seasonally flooded by acidic, nutrient-poor black-water rivers (igapós^31,32^). Finally, both types of flooded forests are more diverse than naturally open areas on nutrient-impoverished sandy soils (campinas^31,33–36^). The drivers of these patterns remain elusive but may be associated with geological processes, soil fertility, inundation gradient, type of water^37^ and also with the size and fragmented distribution of these “smaller vegetation types” on which the colonization of species may be in part attributed to chance^38,39^. However, such patterns could in principle be specific to plants and vertebrates. Other taxa, such as fungi, bacteria and other micro-organisms could display different diversity patterns. Indeed, in a previous study using part of our data, we found different patterns for micro-organismal richness among Amazonian habitat types^40^, but a similar pattern of higher terra-firme diversity than campina diversity was found for fungi in Colombian Amazonia^41^. The contrasting patterns between micro- and macro-organisms may have major implications for our understanding of general diversity patterns and for conservation.

**Figure 1:**
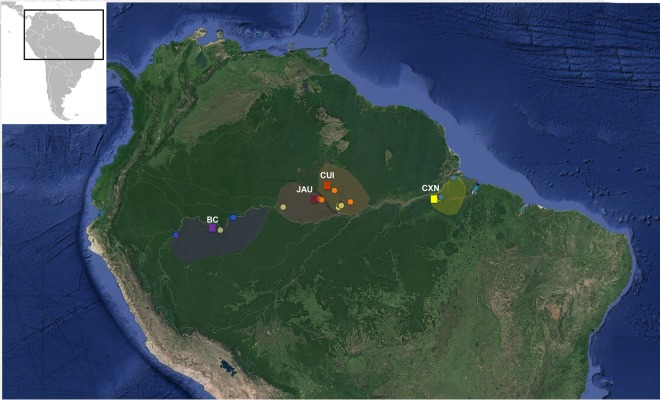
Map of sampling localities. Circles represent plots pertaining to the Amazon Tree Diversity Network (ATDN) used in this study, which represent different forest types: igapós (orange), várzeas (blue) and terra-firme (green). The semi-transparent polygons show the interfluves from which those plots were selected. Squares represent the locations of the metabarcoding sampling that were compared to the ATDN data. In each locality, we sampled different habitats: in Benjamin Constant (BC) we sampled terra-firme, igapós and várzeas; in Jaú (JAU) and Cuieras (CUI) we sampled terra-firme, campinas and igapós. At each of the three localities we sampled nine plots. In Caxiuanã we sampled terra-firme, campinas, várzeas and igapós, totaling 12 plots. The map was contructed with Qgis v.3.6.2^96^.

In this study, we test whether patterns of tree and avian species richness are similar to those found in Operational Taxonomic Units (OTU^42^) mainly targeting micro-organisms. For this purpose, we compare OTU richness generated from environmental sequencing in four Amazonian localities, with richness estimates from existing taxonomic inventories for trees and birds in the vicinity (Fig. 1). For the OTU analyses, we examine three different sample types (soil, litter and insect bulk samples) and three sequence markers (the ribosomal 16S, 18S and the mitochondrial COI, which target prokaryotes, eukaryotes and metazoans, respectively). We test if large-scale diversity patterns known from plants and birds (increasing richness from east-to-west and from campinas to flooded forests and to terra-firme forests) can be recovered with our OTU and inventory data. If OTUs and traditional taxonomic species richness show approximately the same diversity patterns, metabarcoding could offer a rapid and cost-effective alternative for biodiversity assessments, without the demand for taxonomic expertise. In that case, the detection and protection of high diversity areas would be facilitated^43–45^, and taxonomists could focus on species descriptions and other important directions of research, rather than spending time on routine identifications. If, however, OTU richness and species richness are decoupled, the idiosyncrasies of each taxonomic group would make generalizations difficult and call into question our current understanding of the distribution of biodiversity in the world’s largest rainforest. Importantly, a rapid and reliable assessment of Amazonian diversity is increasingly crucial, as deforestation rates are currently escalating to alarmingly high levels^46^.

## Results

**Figure 2:**
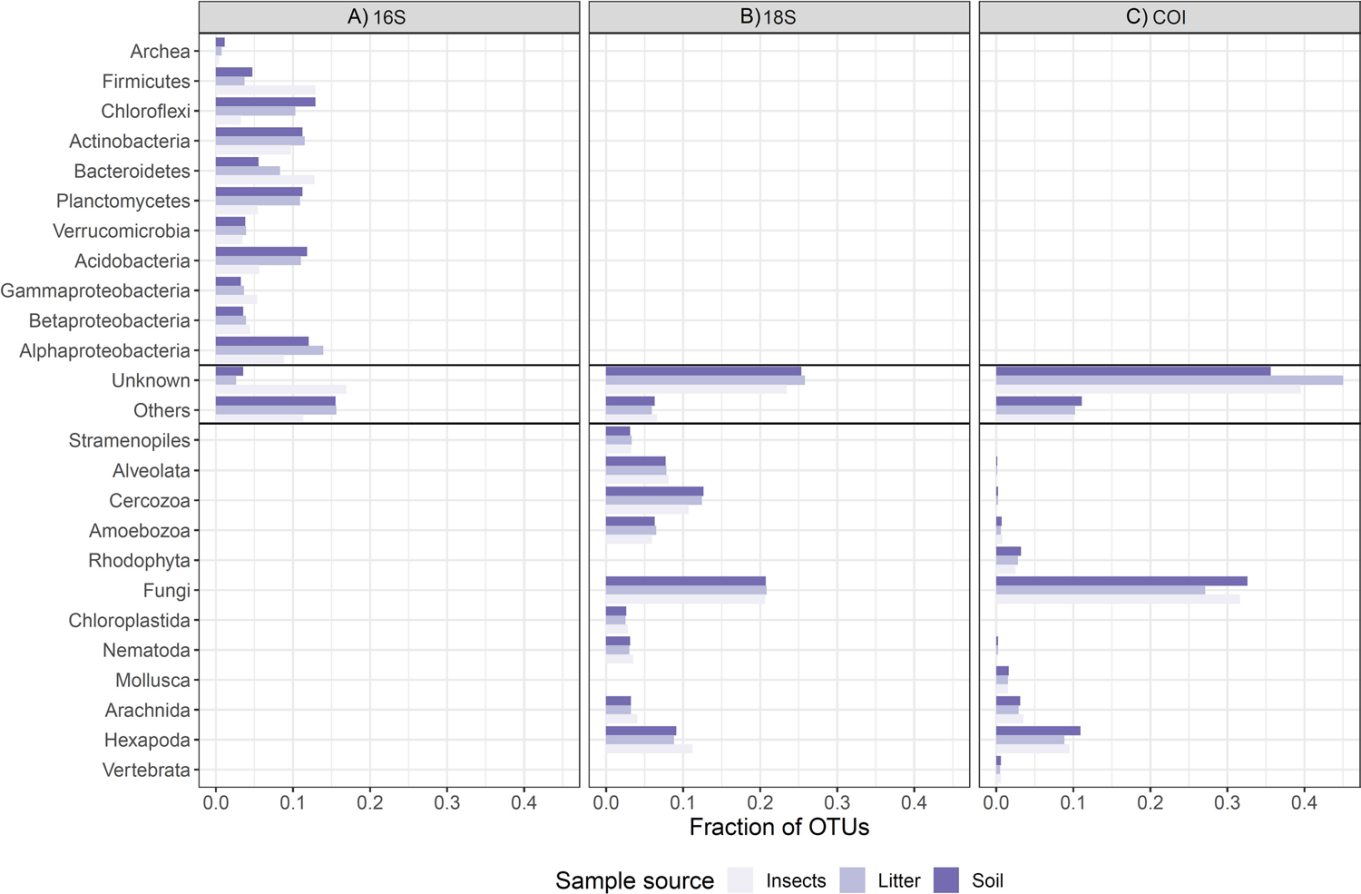
Taxonomic composition of OTU communities. The plots show the breakdown of OTUs into taxonomic groups from (**A**) 16S, (**B**) 18S and (**C**) COI, respectively, coloured by sample type. There is no clear taxonomic variation between soil and litter samples other than some variation in the taxonomic composition for insect samples in the 16S and 18S data sets.

After rarefaction, we obtained a total of 15,563 OTUs for 16S; 17,017 for 18S; and 14,964 for COI (see Supplementary Table S1 for the DNA concentration, number of reads, number of OTUs and Shannon estimate for each plot). The taxa with the highest number of identified OTUs across all samples were: Alphaproteobacteria (15%), Acidobacteria (10%), Planctomycetes (10%), Bacteroidetes (10%), Actinobacteria (10%) and Chloroflexi (10%) (Fig. 2A) for prokaryotes (the 16S marker); and Fungi (20%, mainly Ascomycota and Basidiomycota), Cercozoa (15%) and Alveolata (10%) (Fig. 2B) for eukaryotes (the 18S marker). For the COI marker, the taxa with the highest number of OTUs were Fungi (30%, mainly Ascomycota and Basidiomycota) followed by Hexapoda (10%; Fig. 2C). The proportion of unclassified OTUs was around 10%, 25% and 40% for 16S, 18S and COI, respectively, reflecting the incompleteness of public databases for these markers, beyond the possible sequence errors/chimeras. The lack of representative sequences is more problematic for COI, since usually this marker is sequenced just for metazoans.

**Figure 3:**
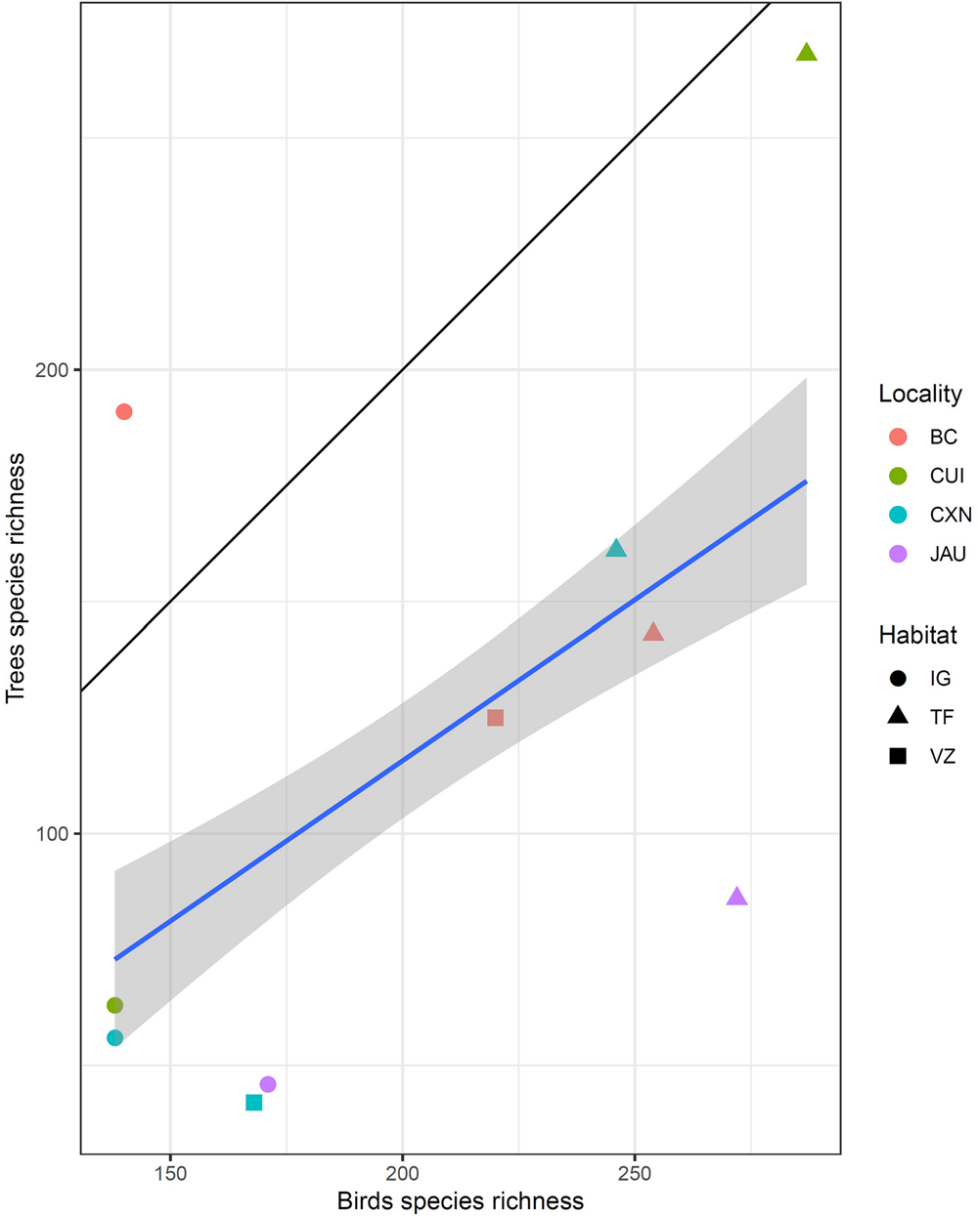
Regression between plot-level species richness of trees and regional species richness of birds for the localities sampled. The thick blue line shows the linear regression with standard error indicated by the shaded area. The thin solid black line shows x = y (perfect correlation). There is a weak but significant relationship between the species richness of these two taxonomic groups (posterior mean = 0.01; p < 0.001). The colour represents the localities: BC = Bejamin Constant, CUI = Cuieras, CXN = Caxiuanã, JAU = Jaú. The symbols represent the habitat type: IG = igapós. TF = terra-firmes, VZ = várzeas.

Regional species richness for birds was poorly related to the average plot-level species richness for trees (posterior mean = 0.01, p < 0.001; Fig. 3). When divided by habitat, the regressions were significant for terra-firme (adjR^2^ = 0.21, p = 0.002) and igapó (adjR^2^ = 0.11, p = 0.03). Only two datapoints were available for várzeas.

**Figure 4:**
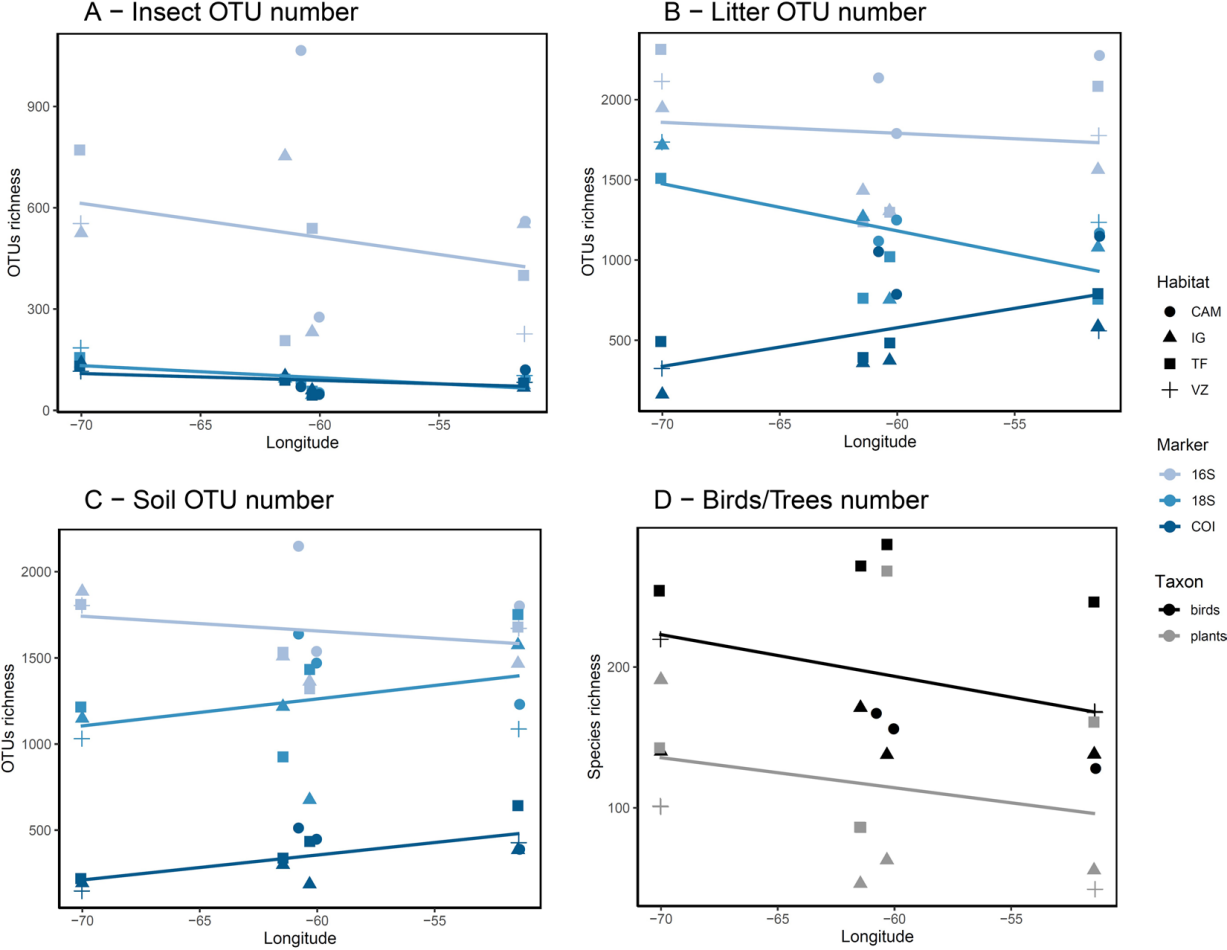
Metabarcoding OTU and species richness of birds and trees per longitude and habitat type. The plots show OTU richness measured from metabarcoding samples of (**A**) insects, (**B**) litter and (**C**) soil. Plot (**D**) shows the known species richness for trees and birds from which those samples were obtained. The colour-coding in A–C indicates marker type and in D the taxonomic group and the symbols indicate habitat type (CAM: campinas, IG: igapó, TF: terra firme and VZ: várzea). The results for A–C indicate that OTU richness varies significantly with location and habitat type, with the highest overall richness obtained from 16S data. For species richness of trees and birds, a consistency between environment richness (TF > VZ > IG > CAM) can be observed, and a west-to-east gradient, as generally expected based on large-scale inventories. For OTUs, an overall pattern with the highest richness in campinas is observed. The west-to-east gradient is observed in general, except for COI litter and 18S and COI soil.

The average species richness of trees (1 ha plot-level), plot-level DNA-based OTU richness and regional bird species richness all decline along a west-to-east gradient (Table 1; Fig. 4). Species and OTU richness were generally decoupled across vegetation types. The species richness of birds and trees showed the richness gradient terra-firme > várzea > igapó > campinas did not show the same gradient among vegetation types, with campinas having the highest richness (Table 1; Fig. 4). The number of species (trees and birds) and DNA-based OTUs per habitat in each locality is available in Supplementary Table S2.

**Table 1:** Mean number of all OTUs (‘meta’; comprising prokaryotes and eukaryotes), OTUs by taxonomic groups and species (‘birds’ and ‘trees’) for locality and habitat.

	Meta (OTUs)	16 S (OTUs)	Protists 18 S (OTUs)	Protists COI (OTUs)	Fungi 18 S (OTUs)	Fungi COI (OTUs)	Metazoa 18 S (OTUs)	Metazoa COI (OTUs)	Birds (regional species)	Trees (average species, 1 ha plot)
**Locality**										
Benjamin Constant	**907**	**1525**	**262**	18	**263**	83	**205**	38	**205**	152
Jaú	813	1336	213	32	212	125	163	65	203	86
Cuieras	714	1074	199	28	200	126	155	61	194	66
Caxiuanã	877	1338	220	**39**	222	**157**	171	**84**	170	**166**
**Habitat**										
Terra-firme	808	1266	214	32	215	127	166	63	**265**	**164**
Várzea	843	1358	234	21	236	106	**187**	56	194	82
Igapó	757	1212	215	19	214	97	170	44	147	89
Campina	**973**	**1511**	**239**	**48**	**241**	**176**	178	**95**	150	N/A

OTUs were divided by the main taxonomic group; 16S comprises mostly bacteria, and 18S and COI were divided into protists, fungi and metazoan. For localities, the mesuared richness shows a gradient from west to east: Benjamin Constant > Jaú > Cuieras > Caxiuanã. For habitat type, the measured richness reflects the order expected based on the literature for macro-organisms: terra firme > várzea > igapó > campinas. The highest richness in each category is highlighted in bold. The patterns are different from our expectations for localities, with Caxiuanã being richer than expected for metabarcoding and trees. For habitats, OTU richness is also different from the expected, but for birds and trees the species richness reflects the previously documented pattern. Tree richness is not reported for campinas since it does not capture the known flora of those habitats and is dominated by other growth forms (e.g., herbs and shrubs).

**Figure 5:**
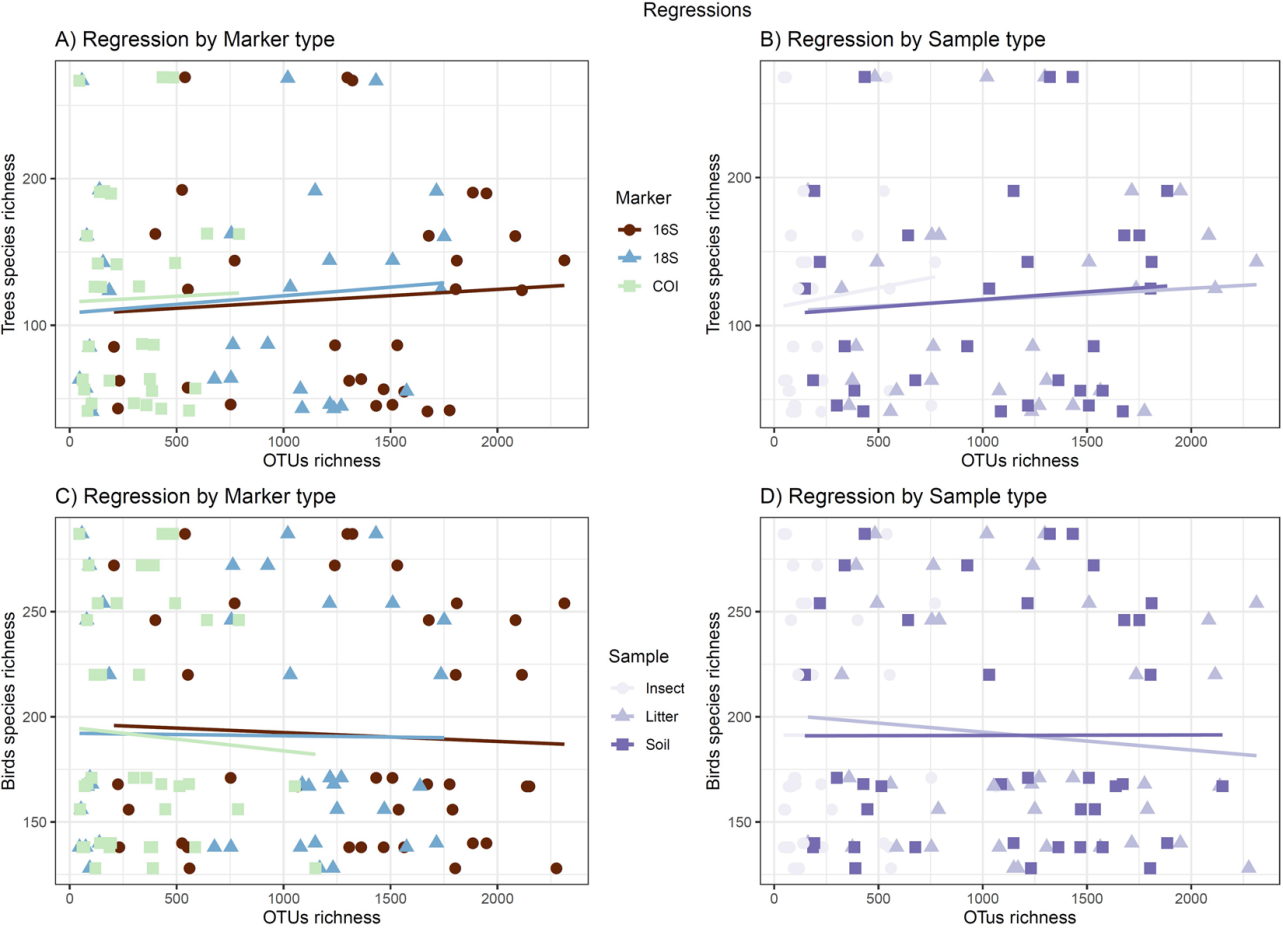
Regression between OTU and species richness. The lines show the regressions between OTUs and tree richness in (**A**, **B**) and between OTU and bird richness in (**C**, **D**). The samples are coloured per marker in (**A, C**) and per sample type in (**B, D**). The vast majority of slopes are positive between OTU and tree richness. However, for birds there is no consistency in the relationship between DNA-based OTU and bird species richness.

The relationship between plot-level DNA-based OTU and plot-level tree and regional bird richness was not significant (posterior mean = 0, p > 0.05 for both tests that were analyzed separately; Table 2). Only the metabarcoding predictors (sample and marker type) were significant in both models (plot-level DNA-based OTU richness *versus* nearby plot-level tree richness and plot-level DNA-based OTU richness *versus* regional birds richness; Table 2). We found the same pattern when we subdivided the metabarcoding data based on taxonomy (prokaryotes, protists, fungi and metazoan; Table S3). The random effects of “locality” and “habitat” type were not significant. We found a significant positive relationship between plot-level DNA-based OTU richness and plot-level species richness of nearby tree plots (eight positive regressions out of nine tests; p = 0.039; Table 3; Fig. 5) when considering a binomial distribution. In contrast, there was no clear relationship between plot-level OTU richness and regional bird species richness (five negative regressions out of nine tests; two-tailed probability 0.51; Table 3; Fig. 5).

**Table 2:** Coefficients for the general linear model fitted in a Bayesian framework using Markov chain Monte Carlo (MCMC) methods for OTU richness against species richness of trees and birds.

Taxon	Effect	Variables	post.mean	l-95% CI	u-95% CI	eff.samp	pMCMC
Trees	Fixed	Richness taxa	0.00	0.00	0.00	693.3	0.464
		Marker 16S	**5.73**	5.47	6.04	1000.00	<0.001
		Marker 18S	**5.00**	4.67	5.28	1000.00	<0.001
		Marker COI	**4.17**	3.88	4.47	1000.00	<0.001
		Sample Litter	**1.80**	1.58	2.02	1136.00	<0.001
		Sample Soil	**1.68**	1.45	1.91	1000.00	<0.001
	Random	Locality	0.14	0.00	0.07	107.5	NA
		Habitat	0.00	0.00	0.00	1000.00	NA
Birds	Fixed	Richness taxa	0.00	0.00	0.00	873.78	0.902
		Marker 16S	**5.81**	5.39	6.20	1000.00	<0.001
		Marker 18S	**5.03**	4.66	5.44	1000.00	<0.001
		Marker COI	**4.29**	3.90	4.69	1000.00	<0.001
		Sample Litter	**1.89**	1.67	2.10	1000.00	<0.001
		Sample Soil	**1.75**	1.53	1.95	1000.00	<0.001
	Random	Locality	0.01	0.00	0.04	338.1	NA
		Habitat	0.01	0.00	0.02	711.6	NA

The model was adjusted with the Poisson family distribution considering taxonomic richness, marker and sample type as fixed effects, and locality and habitat type as random effects. For trees and birds, the taxonomic richness is not significant, whereas the marker and sample type are. Significant values of the post mean of the coefficients (at p < 0.05) are shown in bold.

**Table 3:** Results for the generalized linear mixed effects models considering each marker and sample type separately.

Taxon	Sample type	16 S	18 S	COI
Trees	Insect	**0.26**	**0.01**	**0.004**
	Litter	**0.17**	**0.13**	*−0.02*
	Soil	**0.09**	**0.38**	**0.18**
Birds	Insect	**0.09**	*−0.08*	*−0.10*
	Litter	*−0.08*	*−0.18*	**0.29**
	Soil	*−0.01*	0.23	**0.32**

For each model, the coefficient is presented. No single regression is significant after Bonferroni correction for multiple tests (p < 0.00275). In order to illustrate the pattern in the sign of the effect, we have given all positive slopes in **bold** and all negative ones in *italics*. It is evident that the vast majority of slopes are positive between OTU and tree richness (8/9 P = 0.039), while there is no consistency for the relationship between OTU and bird richness (4/5 n.s.).

## Discussion

Our study indicates that OTU and species richness shows a declining west-to-east diversity gradient, yet the biodiversity patterns of macro- and DNA-based OTUs are largely decoupled across Amazonia. We found no relationship between DNA-based OTU richness estimated from metabarcoding of environmental samples and species richness estimated from previous field inventories. These results suggest that at the regional scale, the diversity distribution of one taxonomic group should not be used as a general proxy for diversity of another, nor as an indication of overall patterns of richness. At small spatial scales, the idiosyncrasies of each taxonomic group and the peculiarities of each environment appear to be more important than general diversity patterns, which differ among organism types.

It is important to acknowledge that we compared data aggregated at different spatial scales and generated using different methods in order to produce the richness estimates used here. In addition, there are differences in the exact locations of the trees surveyed and the metabarcoding plots sampled for this study. These considerations make a direct comparison of richness challenging and worth further exploration by future studies based on primary inventories. However, our primary aim was to assess correlations between *proxies* of species richness. This means that despite these challenges, if the regional-scale processes are important (locality, habitat type), the levels of alpha diversity should increase as a function of the source pool (unsaturated type I relation^47,48^). Therefore, if the west-to-east gradient or habitat differences hold true for all samples, a positive and significant relationship should be found across our data sets. If not, this would suggest that other factors may be more important in determining richness from local to regional scales.

For prokaryotes, diversity is often high in pastures and agricultural fields, which generally have low animal and plant diversity at the local to regional scale^49–53^. However, some bacterial groups, such as the Alphaproteobacteria and Planctomycetes^53^, are more diverse in undisturbed forests. Both of these groups were abundant in our samples, accounting for 35% of our 16S data (Fig. 2A). As a result, when looking for general patterns of richness in bacteria, a negative correlation with trees and birds could be expected, but these effects could be masked by other groups that are positively correlated with macro-organisms, as is the case in Alphaproteobacteria and Planctomycetes.

Patterns of diversity can be distinct for different taxonomic groups, and the wide taxonomic range of metabarcoding studies can mask taxon-specific patterns. Furthermore, different markers target different species and may have added some noise in our analysis. For instance, for fungi in litter samples, 18S and COI displayed the opposite pattern (Fig. S1). Previous studies have reported a decoupling between fungi and plant diversity worldwide^11^ whereas others have found a positive relationship^13,41^ and a similar community turnover^54^. For other groups, such as insects, diversity is often positively correlated with plant diversity^55,56^. Additionally, soil protists can have similar biogeographic patterns to macro-organisms in lowland Neotropical rainforests^12^, which is expected to have a positive effect in the regression of protist OTUs and tree and bird species richness. Our data showed similar patterns overall for metazoans, fungi and protists for these same markers (Table S2, Fig. S1). However, our results highlight the need for further exploration of biotic interactions and diversity metrics, as contrasting results can be found within the same taxonomic groups (e.g. fungi sequenced with 18S and COI, Fig. S1).

A west-to-east decline in diversity has repeatedly been documented in birds^57,58^ and plants^20,21,57^ for Amazonia and is also partly reflected in our metabarcoding data, other than for the easternmost locality (Table 1). A positive correlation with this diversity should be found if all groups shared the same overall diversity pattern due only to the same abiotic conditions (e.g. moisture^28^, nutrient levels^59^ or geology^26^), yet regional and local deviations appear idiosyncratic among taxa. For instance, the combined data from the Amazon Tree Diversity Network across the entire Amazon basin cleary show a west-to-east diversity gradient, but contain multiple outliers in the eastern part of the Negro River close to the Cuieiras area surveyed here^21^. This is consistent with the observed higher-than-expected tree richness in terra-firme from Cuieras as revealed from our data (Fig. 4D) and this may have affected the results of our regressions due to our limited sampling. In addition, Benjamin Constant has the poorest bird inventories, possibly resulting in underestimated richness for this area in our data.

By adding more data and analyses to our previous study^40^, we could provide further evidence that the plot-level DNA-based OTU richness gradient differs from the plot-level tree species richness and from the regional bird species richness across vegetation types. The general richness pattern for vertebrates and plants, also reported here with our bird and tree data, is: terra-firme > várzea > igapó > campina^21,30–36^. However, we found that campinas make up the richest habitat in our OTUs data (Table 1, Fig. 4). This habitat is usually considered less diverse for macro-organisms than more forested habitats in Amazonia, such as terra-firme and flooded forests^18,20,21,33,36^, a relationship confirmed for Colombian Amazonian fungi^41^. Previous studies have reported on the importance of campinas for beta-diversity^36^, but these habitats have long been considered species-poor environments^60^. In contrast, our results suggest that these environments may be hyperdiverse for microbial diversity (Fig. 4A–C). However, we note that campinas have an insular distribution in Amazonia, being surrounded by a “sea” of terra-firme forests^61,62^. OTU diversity in these patches could, potentially, be over-estimated due to DNA transported from nearby forest species, for instance through leaves, fungal spores and other debris^63^. This effect will be hard to test, but it is important to stress that the community composition of campinas was significantly different from the other habitats^40,64^ and there is a rich micro-organismal community that is genuinely from campinas.

The different spatial scales for our analyses – plots of 28 m of radius for metabarcoding data, 1 ha plots for trees and species pools in the interfluvia for birds, influences our species richness comparison. However, within each taxonomic group, the species richness should be consistent across these scales if the west-to-east and habitat gradients are the dominating factors explaining the richness gradient. The outliers in our data (e.g. tree richness in Cuieras and OTU richness in campinas) may have had the strongest effect in the general regression between the OTUs and species richness for birds and for trees. For trees, the pattern we recovered reflects outliers already identified in a previous study^21^. These considerations suggest that even with the different spatial scales used here and in other studies, if the west-to-east gradient was the strongest factor explaining diversity, it should produce a positive correlation. However, the outliers showed that the specifity of localities affected the general pattern.

There are still numerous uncertainties in the underlying biodiversity data and in our ability to generalize overall diversity patterns and identify their main determinants from local to regional scales. We therefore recommend the further validation of the patterns reported here through the generation and analysis of independent data, sampled under standardised conditions for multiple organism groups. With a standardized protocol and additional analyses, such as, for example, that of the metatranscriptome^65^ to target only metabolically active organisms, it will be possible to avert these shortcomings and to draw stronger conclusions on species interactions^66,67^, abiotic diversity drivers^64,68^ and above-ground and below-ground feedback^69^.

A recent global study comparing below-ground organisms (bacteria, fungi and mesofauna) with above-ground organisms (mammal, birds, amphibians and vascular plants) found a diversity mismatch of 27%^9^. The findings from this and previous studies that micro- and macro-organismal diversity are often decoupled has important implications for conservation. It is genuinely worrying in the context of bioversity loss^70^, since a large proportion of the world’s biodiversity may be lost without notice, particularly in Amazonia^46^. Micro-organisms are essential for ecosystem functioning, as they constitute the majority of the diversity of any ecosystem. As highlighted by O’Malley & Dupré^17^, the excessive focus on macro-organisms may have distorted our understanding of general patterns of biodiversity. There is therefore a danger that conservation strategies may be inadequate, if their primary focus is to maintain ecosystem functionality and the biotic interactions^71^.

## Conclusions

In this study, we found that other than displaying a declining west-to-east gradient at large spatial scale, species richness patterns are not consistent across taxa in Amazonia. In particular, patterns in the diversity of micro-organisms (which comprise the bulk of the total diversity) differ strongly from patterns in birds and plants, particulary in connection with habitat type. Furthermore, we found large differences in species richness and diversity patterns between i) metabarcoding of environmental samples and nearby taxonomic inventories, and ii) different genetic markers used for DNA barcoding. Importantly, our results suggest that diversity patterns differ considerably among taxonomic groups, making the use of single taxa as a proxy for total diversity problematic, especially for conservation purposes. This study highlights the importance of integrative and data-rich approaches to studying and describing diversity.

## Material and Methods

### Study areas

We sampled four localities across a west-to-east transect in Brazilian Amazonia (Fig. 1^40^). These areas include: Benjamin Constant (a municipality which is the westernmost locality in our sampling scheme, located south of the Amazon river); Jaú (a national park in central Amazonia situated west of the Negro river and north of the Amazon river); Cuieras (a biological reserve east of the Negro river and north of the Amazon river); and Caxiuanã (the easternmost locality in our sampling: a national forest situated south of the Amazon river; Fig. 1). We chose these localities to maximize geographic distance and to cover all major vegetation types, i.e. terra-firme, várzeas, igapós and campinas (see ref. ^40^ for a more detailed description of the locations surveyed).

### Sampling of metabarcoding data

We collected mineral soil, litter (the organic matter above the mineral soil) and insects in three plots in each major vegetation type present at each locality (3 to 4 depending on the locality; see ref. ^40^ for more details) in November 2015. First, we installed a SLAM trap in the middle of each plot. SLAM traps are dome-shaped, tent-like insect traps made of fine mesh-netting, widely used in entomological studies and aimed at capturing strong-flying insects that typically fly upwards after hitting a fine-scale net (e.g. wasps, mosquitos and butterflies). These insects were ultimately trapped in a bottle filled with ethanol at 96% concentration. The traps were kept open for 24 hours in each plot. After capture, the insects were preserved in a clean plastic bottle with new 96% ethanol until DNA extraction.

We sampled soils and litter following Tedersoo *et al*.^11^ to minimize information loss while keeping comparability between this and other large-scale studies. First, 20 trees were randomly selected within a 28 m radius of each SLAM trap. To reduce the risk of contamination, we wore gloves and a nose-and-mouth mask and replaced the gloves between each sampled tree. We sampled litter and soil cores in opposite directions of each selected tree. In total, 40 soil and 40 litter samples were collected per plot. The soil and litter samples were subsequently pooled into one combined soil and one combined litter sample for each plot. The litter consisted of all organic material above the mineral soil and varied from 0–50 cm in thickness. We then collected soil in the same places, with the samples taken from the top 5 cm of the mineral soil using a metal probe with a 2.5 cm diameter. The soil probe was sterilized with fire after collecting soil from both sides of each tree to prevent cross-contamination between samples. The samples were stored in plastic bags with the same weight of sterilized white silica gel (14 mm silica grain size). The silica was pre-treated for two minutes in a microwave oven (800 W) and exposed to 15 min of UV light to prevent contamination in our samples from any micro-organisms present in the silica. All plots were tagged with GPS coordinates. All dry soil, litter samples and ethanol insect samples were processed at the University of Gothenburg, Sweden. For more details of the collection protocol, see ref. ^43^.

### DNA extraction

For soil, 10 g (dry weight) of each sample and 15 ml of each litter sample (corresponding to 3–10 g of dry weight litter, depending on texture and composition of each sample) and a negative control were processed for total DNA extraction using the PowerMax® Soil DNA Isolation Kit (MO BIO Laboratories), according to the manufacturer’s instructions (see details in ref. ^40^). For insects, we followed the non-destructive protocol described in Aljanabi and Martinez^72^, we also included a negative control for insect extractions.

### PCR Amplification

We used three genetic markers to target different organisms: 16S for prokaryotes, 18S and COI for eukaryotes in general. For amplification of ribosomal small subunit (SSU) 18S rRNA in soil and litter samples, we targeted the V7 region of the gene using the forward and reverse primers (5′-TTTGTCTGSTTAATTSCG-3′) and (5′-TCACAGACCTGTTATTGC-3′) designed by Guardiola *et al*.^73^ to yield 100 to 110 base pair (bp) fragments (see details in ref. ^27^). For the ribosomal small subunit (SSU) 16S rRNA, we targeted the V3–V4 region (~460 bases) of the 16S rRNA gene using the forward primer (5′-CCTACGGGNGGCWGCAG-3′) and reverse primer (5′-GACTACHVGGGTATCTAATCC-3′) from Klindworth *et al*.^74^. For the cytochrome c oxidase subunit I mitochondrial gene (COI), we amplified a region of ~313 bases using an internal forward primer (5′- GGWACWGGWTGAACWGTWTAYCCYCC-3′^75^) and the COI degenerate reverse primer (5′-TAAACTTCAGGGTGACCAAARAAYCA-3′^76^). Amplification and sequencing were carried out by Macrogen (Republic of Korea) following standard protocols using the Illumina MiSeq. 2 × 250 (18S) and 2 × 300 (16S and COI) platform, including the negative control to check possible sequences errors and cross-sample contaminations^77^. Part of the data presented here has already been published. The soil and litter data for 16S and 18S were already analysed in previous studies^40,64^. Soil for COI and insect samples for the three markers were previously analysed in Benjamin Constant^43^. Here we present new data for COI for litter (all data), and COI for soil; as well as16S, 18S and COI for insects for Jaú, Cuieras and Caxiuanã. All raw sequences are available in GenBank under Bioproject PRJNA464362.

### Sequence analyses and taxonomic assessments

We used the USEARCH/UPARSE v9.0.2132 Illumina paired reads pipeline^78^ to merge the paired sequences with a maximum of five mismatches allowed, truncate by the length (80 bp for 18S, 400 bp for 16S and 290 bp for COI), filter sequence reads for quality and discard reads with >1 total expected errors for all bases in the read after truncation, de-replicate and sort reads by abundance, infer OTUs by 97% of similarity and remove singletons. We filtered the data to discard artificial sequences (e.g. chimeras), and we clustered sequences into OTUs at a minimum similarity of 97% using a “greedy” algorithm that performs chimera filtering and OTU clustering simultaneously^78^. We address all OTUs registered in the negative controls (18S = 595 OTUs, 16S = 379 OTUS, negative control fail in sequencing for COI) and excluded them from our data sets (Tables S4. and S5). For 16S and 18S data, we used SILVA 1.3^79^ for assessment of the taxonomic composition of the OTUs, using a representative sequence from each OTU as query sequence and the SINA v1.2.10 reference data for ARB SVN (revision 21008^80^) for local BLAST searches^81^ of both markers. As reference COI data, we used all COI sequences deposited in GenBank until August 2018^82^ in our BLAST searches. All searches were conducted with the same criterion: a minimum 80% similarity and an e-value of 0.001.

### Compilation of taxonomic data

We compared the OTU diversity estimated from our environmental samples with morphology-based taxonomic estimates of species richness for trees and birds. For trees, we used the data from the Amazon Tree Diversity Network (http://atdn.myspecies.info/). That project links plots across all Amazonia from different vegetation types, where a full inventory was made of all free-standing trees up to 10 cm in diameter at breast height (dbh). Trees were identified to the level of species or morphospecies. We compiled the mean richness of tree species in all 1-ha plots within each ecosystem type and interfluvial for which we had metabarcoding data (Fig. 1). For two plots that had an area of 1.3 ha, we estimated the number of individuals expected in 1 ha (number of individuals / 1.3). We then rarefied the plot by the number of expected species using the “rarefy” function in the package vegan v. 2.4–3^83^ in R v3.3.2^84^. Since trees are only a minor component of the vegetation in campinas^85^, we considered them a poor proxy for plant diversity in such plots. We therefore excluded campinas in the analyses of the relationship between trees and OTU richness.

For birds, we used published compilations for our study sites whenever available. This was the case for Jaú National Park^33,86^, and Caxiuanã National Forest^87^. For Cuieiras, we used data from Manaus, a well-studied nearby locality^88^ that is situated in the same interfluvium area and should therefore have a very similar species pool. For Benjamin Constant, which lacks available published sources, we created a hypothetical species list based on data from the Global Biodiversity Information Facility (GBIF, www.gbif.org) (Fig. 1), which was carefully validated by an expert on Amazonian avian distribution patterns (author’s acronym, L.N.). For each locality, L.N. classified all species by habitat type(s) based on his field experience, complemented by published sources. Bird species lists and habitat classification are available as Supplementary material (Table S4).

### Statistical analyses

Since the number of observed OTUs was dependent on the number of reads, we first rarefied all samples to the lowest number of reads obtained from any one plot (23,132 for 16S, 25,144 for 18S and 25,280 for COI; Fig. S1) using the function “rarefy” of the R package vegan v. 2.5–4^83^. For 18S, we discarded one sample (“SJAUTFP1”) with a very low number of reads (1,395). As the rarefaction and richness estimates could be biased by rare OTUs^89^, we also calculated OTU diversity of order q = 1, which is equivalent to the exponential of the Shannon entropy^90^. We did so by transforming the read counts using the “varianceStabilizingTransformation” function in DESeq. 2^91^ as suggested by McMurdie & Holmes^92^. This transformation normalizes the count data with respect to sample size (number of reads in each sample) and variances, based on fitted dispersion-mean relationships^91^. As the results were virtually identical (Pearson correlation > 0.99 for all data sets) we used the richness based on rarefaction of OTUs for further analyses, since we had no abundance data for birds and just richness measurements was possible. The results of both richness by rarefaction and Shannon estimated are presented in Table S2. As we had three plots in each environment at each locality, we used the mean of the three plots for each environment at each locality.

We tested the relationship between the mean species richness per habitat type of trees and birds by fitting a generalized linear mixed effects model in a Bayesian framework, using Markov chain Monte Carlo (MCMC) methods implemented in the R package MCMCglmm v.2.28^93^. We used this method to control for nested sampling^94^, because our plots are nested in the habitat types and we pooled all of them into one regression, but they might differ in their intercept. In this case a mixed effects model would be better suited, since it allows different intercepts. To test the relationship between OTU richness and species richness, we also fitted generalized linear mixed effects models using the OTU richness as the response variable and the genetic marker (16S, 18S and COI), sample type (soil, litter and insects) and tree or bird richness as explanatory variables. We used the Poisson family distribution in the model and considered locality and habitat type as random effects in both analyses. Because the organisms’ body size^95^ and/or the taxonomic reponses to environmental conditions^10^ could affect the diversity patterns, we also divided our data into 16S that comprises mostly bacteria and divided our 18S and COI data between protists, fungi and metazoan, and fitted generalized linear mixed effects models separately for each data sets.

To further assess whether there was any tendency for a positive or negative relationship between OTU and taxonomic diversity, we fitted separate generalized linear models between each OTU richness variable (3 markers and 3 sample types, totaling 9 response variables) against the tree and bird richness separately. We assessed the relationship of these variables based on a two-tailed binomial distribution only focusing on the sign of the relationship. The null expectation is that ~50% of all relationships would be positive and ~50% would be negative if there were no underlying patterns and the relationships were independent of each other. An overabundance of either positive or negative relationships can therefore be seen as a significant deviation from the null-expectation. In our analyses, we carried out a total of nine tests (OTU richness for 3 markers and 3 sample types). The combined probability of achieving 0, 1, 8 or 9 positive outcomes out of nine attempts if both positive and negative relationships are equally likely is 0.039. We therefore considered a relationship where 0, 1, 8 or 9 of the slopes were positive as significant.

### Permit(s)

Collection permits for this study were granted by the Brazilian authorities ICMBio (registration number 48185–2) and IBAMA (registration number 127341). The SisGen registration number is A8A9AB7.

## Supplementary information

Supplementary Information
