# Supplementary material for: The pitfalls of biodiversity proxies: Differences in richness patterns of birds, trees and understudied diversity across Amazonia
Source: Sci Rep. 2019 Dec 16;9:19205. doi: 10.1038/s41598-019-55490-3 (PMC6915760; doi:10.1038/s41598-019-55490-3)
Supplement: Supplementary file 1 — Supplementary Information [file 41598_2019_55490_MOESM1_ESM.pdf]

SUPPLEMENTARY MATERIAL FOR:

The pitfalls of biodiversity proxies: Differences in richness patterns of birds, trees and understudied diversity across Amazonia

Running title: Birds and trees *vs.* understudied diversity in Amazonia

Ritter, Camila D*.[1,2,3], Faurby, Søren [2,3], Bennett, Dominic J. [2,3], Naka, Luciano N.[4], ter Steege, Hans[5,6], Zizka, Alexander[7], Haenel, Quiterie[8], Nilsson, R. Henrik [2,3£], Antonelli, Alexandre[2,3,9£]

[1] Department of Eukaryotic Microbiology, University of Duisburg-Essen, Universitätsstrasse 5 S05 R04 H83 D-45141 Essen, Germany

[2] Gothenburg Global Biodiversity Centre, Box 461, SE-405 30 Göteborg, Sweden

[3] Department of Biological and Environmental Sciences, University of Gothenburg, Box 463, SE-405 30 Göteborg, Sweden

[4] Laboratório de Ornitologia, Departamento de Zoologia, Universidade Federal de Pernambuco, Recife, PE, Brazil.

[5] Naturalis Biodiversity Center, Leiden, Netherlands.

[6] Systems Ecology, Free University, Amsterdam.

[7] German Centre for Integrative Biodiversity Research (iDiv) Halle-Jena-Leipzig, Deutscher Platz 5e, 04103 Leipzig.

[8] Zoological Institute, University of Basel, Vesalgasse 1. CH-4051 Basel, Switzerland.

[9] Royal Botanic Gardens, Kew, TW9 3AE, Richmond, Surrey, UK

*Corresponding author: Camila D. Ritter, kmicaduarte@gmail.com. Phone: +55 48991434597. Postal address: University of Duisburg-Essen, Universitätsstrasse 5 - S05 R04 H83. D-45141 Essen, Germany.

[£]Both authors have contributed equally to this paper.

Table of Contents

Table S1 3
Table S2 8
Table S3 10
Table S4. 13
Figure S1 44
Figure S2 46

Table S1: Initial DNA concentration (DNA conc.), number of reads (reads), operational taxonomic units (N Otus), richness rarefied (Raref) with the standard error (SE) and Shannon estimation per sample. The codes in the plot mean: I = Insects samples; L = Litter samples; S = Soil samples. Following by localities: BC = Benjamin Constant; CUI = Cuieras reserve; CXN = Caxiuanã National Forest; JAU = Jaú National Park. Following by habitas: CAM = campina; IG = igapó; TF = terra-firme; and VZ = várzea. A few insect samples did not yield enough DNA and were excluded from the analyses. The negative control had an intial DNA concetration of 0.48 for 18S and 0.12 for 16S and we showed the mean in the table. For COI the initial concentration was 0.07 and it fail in the sequencing.

		16S					18S					COI				
**Plot**	**DNA conc.**	**Reads**	**N_Otus**	**Raref**	**SE**	**Shannon**	**Reads**	**N_Otus**	**Raref**	**SE**	**Shannon**	**Reads**	**N_Otus**	**Raref**	**SE**	**Shannon**
IBCIGP1	3	68897	534	444	7	1502	75930	150	168	2	433	93941	137	134	2	715
IBCIGP2	32	74771	408	347	5	1026	81587	129	130	3	376	61543	160	161	0	825
IBCIGP3	3	67133	632	538	7	1627	82341	137	135	3	430	83571	125	123	1	680
IBCTFP1	124	38712	1296	1177	9	3484	72216	102	110	0	311	85114	108	108	1	579
IBCTFP2	4	86792	552	481	6	1285	75828	204	231	3	575	86813	115	114	1	633
IBCTFP3	1	65680	466	442	4	990	71414	163	180	3	612	80484	171	168	2	896
IBCVZP1	16	74731	411	365	4	987	82702	158	182	0	463	86653	134	132	1	690
IBCVZP2	22	71574	872	764	7	1976	55776	212	230	0	630	88774	114	109	2	572
IBCVZP3	23	78680	376	338	4	961	65027	186	216	0	602	71931	100	100	1	531
ICUICAMP1	106	89675	182	162	3	481	65544	54	62	0	172	99737	48	48	1	255
ICUICAMP2	1	88547	370	324	4	896	68445	52	56	3	324	84811	48	46	1	288
ICUIIGP1	3	85296	158	149	3	413	58335	37	46	0	153	88243	52	50	1	282
ICUIIGP2	137	75684	148	131	3	434	61990	24	30	2	93	87069	44	43	1	234
ICUIIGP3	2	80812	391	352	4	826	60522	78	89	0	271	87250	84	82	1	441
ICUITFP1	31	80453	632	573	5	1412	68978	69	69	3	242	85944	44	43	1	242
ICUITFP2	2	37484	140	131	3	378	67565	47	54	1	158	84575	37	36	1	199
ICUITFP3	21	49016	844	710	9	2312	63285	54	62	0	167	80571	54	52	1	308
ICXNCAMP1	50	63861	863	780	7	2049	69876	96	99	2	299	88029	147	146	1	810
ICXNCAMP2	51	85235	539	491	5	1248	66941	77	83	0	202	87567	88	86	1	459
ICXNCAMP3	12	71883	279	232	5	736	72718	110	121	0	348	92573	125	124	1	680
ICXNIGP1	14	79660	1061	966	7	2382	80087	88	85	4	314	71680	65	66	1	366

ICXNIGP2	10	75335	168	134	4	480	69134	75	94	0	263	94693	88	87	1	478
ICXNIGP3	2	66840	426	393	4	943	90100	63	76	1	207	98869	50	48	1	330
ICXNTFP1	2	70526	322	293	3	818	67994	98	107	2	275	77013	113	112	1	564
ICXNTFP3	2	66027	478	446	4	1117	71787	62	75	1	225	88315	49	47	1	241
ICXNVZP1	299	64199	104	81	3	312	62278	134	136	2	355	83580	81	81	1	396
ICXNVZP2	6	57762	301	221	6	914	80081	108	110	3	298	83008	108	104	2	649
ICXNVZP3	7	66020	274	243	4	667	73239	68	73	1	234	75318	60	59	1	347
IJAUCAMP1	39	59612	2505	1897	17	6293	66230	86	93	0	273	85110	76	75	1	406
IJAUCAMP2	68	74219	139	114	3	389	66627	73	82	0	227	84428	63	61	1	327
IJAUCAMP3	39	57294	555	483	6	1311	61838	86	94	0	265	78348	70	69	1	415
IJAUIGP1	213	51894	874	805	7	2014	75884	98	106	0	318	76955	115	114	1	628
IJAUIGP3	61	58468	632	584	5	1336	68120	91	105	0	329	81781	88	86	1	469
IJAUTFP1	7	73281	87	67	3	299	57865	63	76	0	271	81662	78	77	1	399
IJAUTFP2	3	61258	366	307	6	948	77283	140	159	2	426	85629	117	114	1	659
IJAUTFP3	45	76427	167	145	3	459	71223	75	87	0	278	81382	71	70	1	370
LBCIGP1	49	38112	1891	1517	15	5380	172935	1636	1012	16	3808	164249	226	193	4	1345
LBCIGP2	22	27120	2066	1906	11	5759	44671	1095	1140	0	2519	84022	53	49	2	286
LBCIGP3	28	31948	1886	1609	14	5219	207677	2413	1720	17	5540	162379	206	182	3	1209
LBCTFP1	58	34316	2421	2047	16	6716	158420	2065	1627	16	4883	157381	435	403	4	2342
LBCTFP2	10	38153	2235	1847	15	6412	36552	1283	1348	0	3036	126604	555	509	5	2830
LBCTFP3	22	46311	2282	1745	17	6660	85405	1181	1189	7	3130	113334	489	457	4	2618
LBCVZP1	82	40298	2105	1659	16	5926	129022	2431	1946	17	5526	129651	422	396	4	2137
LBCVZP2	6	41519	2201	1764	16	6206	82741	1173	1212	5	3010	144006	329	305	3	1787
LBCVZP3	23	57338	2040	1422	17	5941	146856	1603	1207	15	3960	149181	222	198	3	1291
LCUICAMP1	16	49446	1986	1557	15	6013	66635	1378	1316	9	3751	133864	827	772	6	4205
LCUICAMP2	3	53386	1534	1195	13	4711	128996	1212	1063	11	3167	135310	599	565	4	3066
LCUICAMP3	6	39462	1851	1594	13	5460	74166	1160	1076	10	3168	121475	935	883	6	4817
LCUIIGP1	22	38001	1373	1200	11	4303	42319	865	919	0	2270	146876	323	283	5	1748
LCUIIGP2	21	55013	1264	945	13	4142	57874	597	620	7	1810	122663	278	263	3	1570
LCUIIGP3	19	48894	1283	1009	12	4084	94111	799	658	10	2341	128309	523	493	4	2846
LCUITFP1	3	45007	1045	833	11	3343	160800	614	644	6	1854	130405	403	359	5	2341

LCUITFP2	4	45104	1781	1474	13	5337	151010	1469	1155	13	4040	136838	836	785	5	4212
LCUITFP3	2	49111	1069	950	8	3135	105148	977	872	10	2905	158046	210	189	3	1146
LCXNCAMP 1	11	37693	2522	2118	16	7166	39247	812	880	0	2237	141585	1513	1402	8	8038
LCXNCAMP 2	7	42884	2076	1707	15	6187	149799	1767	1272	16	4393	148688	1151	1076	6	5908
LCXNCAMP 3	15	40517	2231	1814	16	6556	70047	927	991	3	2479	110372	784	739	5	4166
LCXNIGP1	28	57740	1591	1162	15	4893	160998	1168	992	12	3230	120959	499	459	5	2720
LCXNIGP2	12	61156	1684	1297	15	5213	193291	1139	859	13	3137	127142	716	639	7	3994
LCXNIGP3	65	40718	1418	1240	11	4365	97691	931	914	9	2544	124124	547	508	5	2881
LCXNTFP1	15	43167	2005	1617	15	6067	35346	463	540	0	1568	115592	646	614	4	3416
LCXNTFP2	6	39320	2069	1691	15	6086	164088	817	747	10	2440	95324	559	522	5	3230
LCXNTFP3	7	40628	2179	1764	16	6430	77026	989	1064	0	2726	156324	1168	1069	7	6159
LCXNVZP1	34	45544	1573	1187	15	4839	87592	730	806	4	2299	65978	411	408	2	2231
LCXNVZP2	44	28533	1805	1654	11	5566	139615	1903	1420	16	4906	103989	618	597	4	3172
LCXNVZP3	44	39870	1950	1570	15	5845	85737	1071	1128	4	2810	114668	645	599	5	3507
LJAUCAMP1	3	42205	2153	1715	16	6151	146209	1297	1038	13	3517	140201	863	803	6	4498
LJAUCAMP2	12	42748	2302	1847	16	6331	93003	1132	956	12	3078	144525	1132	1047	7	5996
LJAUCAMP3	14	37009	1957	1650	14	5787	67975	926	878	9	2628	171953	1164	1043	8	6159
LJAUIGP1	20	45754	1450	1186	13	4533	102429	1532	1373	12	3738	143615	440	395	5	2334
LJAUIGP2	25	50707	1409	1058	14	4379	65794	786	878	0	2108	132217	198	162	4	1174
LJAUIGP3	87	48273	1440	1145	13	4419	96687	1493	1263	13	3651	98574	440	416	4	2338
LJAUTFP1	71	46275	1345	1077	12	4281	120497	914	991	0	2474	114352	571	533	5	3039
LJAUTFP2	24	54154	1442	1084	13	4579	66441	972	1027	0	2474	115352	399	363	5	2099
LJAUTFP3	21	41630	932	725	11	3311	104009	402	479	0	1528	116028	210	190	4	1210
SBCIGP1	3	33644	1768	1566	12	4962	116470	1260	1092	11	3149	167774	164	130	4	967
SBCIGP2	5	39217	1969	1619	14	5728	86427	1134	1118	8	2855	153486	179	155	3	991
SBCIGP3	5	25600	1919	1795	10	5439	69140	1050	1107	0	2580	125087	235	215	3	1230
SBCTFP1	8	31415	1866	1653	12	5485	74849	1113	1147	4	2801	141382	253	231	3	1413
SBCTFP2	6	47660	1916	1530	15	5679	91447	1212	1270	0	2940	153141	194	175	3	1107

SBCTFP3	11	35823	1647	1407	12	4969	141497	1321	1190	11	3324	127348	209	187	3	1222
SBCVZP1	8	31394	1710	1525	12	5015	148969	866	903	6	2551	112421	145	129	3	863
SBCVZP2	6	31022	1893	1698	12	5453	118772	1134	1136	7	2776	113498	151	140	3	909
SBCVZP3	6	33078	1813	1598	12	5363	130277	1092	1094	7	2912	125349	145	126	3	874
SCUICAMP1	4	38131	1762	1496	13	5185	110580	1648	1460	12	4008	143217	497	450	5	2549
SCUICAMP2	1	43648	1098	994	8	3225	122405	984	809	11	3021	150214	199	176	3	1100
SCUICAMP3	14	50202	1754	1423	14	5369	134945	1781	1341	15	4429	121301	645	600	5	3406
SCUIIGP1	25	46288	1413	1172	12	4370	93767	844	677	12	2212	156313	194	167	4	1135
SCUIIGP2	18	51324	1393	1096	13	4348	53730	547	620	0	1827	129216	186	159	4	1149
SCUIIGP3	7	48652	1282	1028	12	4018	120882	640	490	10	1900	155369	179	154	4	1105
SCUITFP1	4	46538	1424	1187	12	4262	219870	1304	1051	11	3335	98445	488	460	4	2598
SCUITFP2	3	46792	1399	1197	11	4073	141855	1701	1392	14	4183	125425	439	406	4	2402
SCUITFP3	3	48322	1144	995	9	3369	232759	1290	941	12	3504	139349	376	341	4	1980
SCXNCAMP 1	6	56399	1827	1375	15	5544	123656	1765	1531	13	4209	141241	359	329	4	1938
SCXNCAMP 2	7	45382	1626	1318	13	4914	127301	1403	1162	13	3608	170502	416	369	5	2103
SCXNCAMP 3	1	53335	1956	1553	15	5677	75409	524	535	7	1670	81256	391	384	2	2134
SCXNIGP1	59	39700	1460	1204	13	4516	136488	1618	1382	13	4273	122275	488	459	4	2585
SCXNIGP2	14	45287	1630	1299	14	4933	120160	1739	1431	14	4443	118787	418	387	4	2135
SCXNIGP3	48	41852	1313	1109	11	4114	97823	1369	1176	12	3424	69000	249	247	2	1307
SCXNTFP1	7	49685	1643	1265	14	5000	74255	1954	1825	11	4546	84661	594	579	4	3014
SCXNTFP2	5	45256	1654	1323	14	4922	184678	1649	1151	15	3939	110653	622	582	5	3284
SCXNTFP3	5	43218	1739	1408	14	5166	113584	1651	1190	16	4233	106346	709	685	4	3722
SCXNVZP1	44	35831	1606	1437	11	4778	123514	908	810	11	2391	151950	341	305	4	1903
SCXNVZP2	102	37799	1771	1498	13	5069	178209	932	1016	0	2576	132365	619	568	5	3190
SCXNVZP3	54	23124	1638	1640	0	4591	69508	1420	1277	12	3363	128346	325	308	3	1537
SJAUCAMP1	6	57803	2234	1716	16	6520	175863	1851	1271	17	4579	119779	579	535	5	3138
SJAUCAMP2	6	30204	2273	2069	12	6402	150597	1835	1439	14	4453	114594	583	559	4	2893
SJAUCAMP3	6	39487	1944	1673	13	5854	131538	1227	1158	10	3096	125323	381	353	4	2017

SJAUIGP1	5	50559	1557	1259	13	4770	75422	1187	1155	8	2982	120062	379	344	4	1948
SJAUIGP2	11	38547	1532	1253	13	4476	102646	927	1005	0	2555	106398	268	250	3	1404
SJAUIGP3	28	46629	1438	1144	13	4378	113176	1537	1276	13	3793	143252	253	218	4	1444
SJAUTFP1	14	44876	1534	1237	13	4690	1393	206	301	0	792	140728	518	483	4	2772
SJAUTFP2	9	4978	139563	1326	13	286	130757	1595	1356	13	1613	1572	286	263	3	1572
SJAUTFP3	6	4432	160957	1133	13	209	25274	978	1085	0	1449	1220	209	170	4	1220
Cnegative	0.3	49594	379	NA	NA	NA	84168	595	NA	NA	NA	NA	NA	NA	NA	NA

Table S2: Operational taxonomic units (OTU) richness average, trees species richness average and bird species count per habitat type in each locaity. BC = Benjamin Constant; CUI = Cuieras reserve; CXN = Caxiuanã National Forest; JAU = Jaú National Park; CAM = campina; IG = igapó; TF = terra-firme; and VZ = várzea. Tree richness is not reported for campinas since it does not capture the known flora of those habitats, which is dominated by other growth forms (e.g., herbs and shrubs). Amphibians (Amph) and mammals (Mam) richness was collected from Jenkins et al. (2013). The metabarcoding data is presented by 16S that comprise Bacteria and Archea (Bact), 18S and COI that were splited in Protists (Prot), Fungi (Fungi) and Metazoan (Metaz). We present the number of OTUs (Rich) and Shannon estimate (Shann).

							16S		18S						COI					
**Sample type**	**Loc**	**Hab**	**Bird**	**Tree**	**Amph**	**Mam**	**Rich Bact**	**Shann Bact**	**Rich Prot**	**Shann Prot**	**Rich Fungi**	**Shann Fungi**	**Rich Metaz**	**Shann Metaz**	**Rich Prot**	**Shann Prot**	**Rich Fungi**	**Shann Fungi**	**Rich Metaz**	**Shann Metaz**
Litter	BC	TF	254	143	118	185	1948	5453	480	1215	477	1215	368	1007	10	88	77	196	29	196
		VZ	220	101	118	185	2313	6596	405	1153	410	1153	300	914	43	376	178	196	90	532
		IG	140	191	118	185	2115	6024	471	1219	466	1219	360	1054	28	233	130	196	61	352
	JAU	TF	272	86	118	185	1790	5395	335	977	330	977	252	814	75	547	271	196	156	906
		IG	171	46	118	185	1307	4176	197	663	198	663	155	600	27	247	156	196	72	439
		CAM	167	NA	118	185	1298	3938	262	855	265	855	221	719	41	344	194	196	92	557
	CUI	TF	287	268	118	185	2276	6636	297	953	298	953	212	764	117	870	377	196	208	1246
		IG	138	63	118	185	1564	4823	260	858	262	858	225	789	44	438	204	196	112	742
		CAM	156	NA	118	185	2084	6195	171	646	175	646	148	640	67	606	259	196	134	841
	CXN	TF	246	161	83	170	1776	5416	311	996	315	996	266	879	41	388	207	196	122	717
		VZ	168	42	83	170	2137	6089	300	932	303	932	214	766	87	658	321	196	172	1053
		IG	138	56	83	170	1433	4444	347	993	343	993	268	845	31	300	146	196	64	387
		CAM	128	NA	82	170	1240	4057	193	638	189	638	153	628	40	349	139	196	70	424
Soil	BC	TF	254	143	118	185	1885	5376	304	811	301	811	253	731	16	122	83	196	31	235
		VZ	220	101	118	185	1810	5378	322	882	327	882	252	797	20	185	97	196	32	203
		IG	140	191	118	185	1805	5277	268	784	272	784	207	693	9	126	63	196	19	133
	JAU	TF	272	86	118	185	1538	4593	400	1104	407	1104	291	920	44	344	173	196	84	512
		IG	171	46	118	185	1363	4245	167	574	168	574	133	530	13	160	92	196	30	198

		CAM	167	NA	118	185	1322	3901	392	1084	392	1084	308	959	40	359	180	196	86	497
	CUI	TF	287	268	118	185	1803	5378	326	910	328	910	244	821	43	381	167	196	80	486
		IG	138	63	118	185	1468	4521	423	1260	422	1260	310	999	29	279	153	196	73	453
		CAM	156	NA	118	185	1679	5029	481	1308	487	1308	354	1029	68	573	227	196	122	722
	CXN	TF	246	161	83	170	1672	4813	284	846	286	846	227	736	35	249	164	196	91	514
		VZ	168	42	83	170	2150	6259	442	1234	449	1234	337	980	44	366	184	196	103	597
		IG	138	56	83	170	1509	4541	320	923	315	923	258	802	25	215	129	196	51	322
		CAM	128	NA	82	170	1532	4700	246	747	243	747	182	647	32	245	122	196	64	395
Insects	BC	TF	254	143	118	185	525	1385	28	94	29	94	26	109	13	88	44	266	25	140
		VZ	220	101	118	185	771	1920	36	125	36	125	36	154	12	70	37	235	25	154
		IG	140	191	118	185	553	1308	44	146	46	146	42	174	8	56	42	241	27	155
	JAU	TF	272	86	118	185	276	689	12	48	11	48	14	70	4	23	24	163	8	48
		IG	171	46	118	185	232	557	14	37	13	37	13	60	7	45	24	143	11	65
		CAM	167	NA	118	185	539	1367	14	41	15	41	12	61	3	20	20	196	8	46
	CUI	TF	287	268	118	185	560	1345	22	62	23	62	22	86	8	55	40	196	25	155
		IG	138	63	118	185	552	1268	21	70	20	70	12	80	6	40	24	196	14	86
		CAM	156	NA	118	185	400	968	17	57	16	57	16	75	8	42	33	196	15	79
	CXN	TF	246	161	83	170	226	631	28	82	28	82	19	71	5	35	32	196	17	101
		VZ	168	42	83	170	1066	2664	21	63	21	63	19	79	6	39	24	196	18	112
		IG	138	56	83	170	753	1675	24	81	24	81	17	101	11	61	27	196	21	132
		CAM	128	NA	82	170	207	568	25	73	24	73	15	92	8	44	35	196	18	105

Table S3 – Coefficients for the general linear model fitted in a Bayesian framework using Markov chain Monte Carlo (MCMC) methods for OTU richness and Shannon diversity divided between prokaryotes, protists, fungi and metazoan richness against species richness of trees and birds. The model was adjusted with the Poisson family distribution considering taxonomic richness, marker and sample type as fixed effects and locality and habitat type as random effects. For trees and birds, the taxonomic richness is not significant, whereas the marker and sample type are. Significant values (at p < 0.05) are shown in bold.

			Richness					Shannon				
**Taxon**	**Effect**	**Variables**	**post.mean**	**l-95% CI**	**u-95% CI**	**eff.samp**	**pMCMC**	**post.mean**	**l-95% CI**	**u-95% CI**	**eff.samp**	**pMCMC**
**Trees versus Prokaryotes (16S)**	**Fix**	Richness taxa	0.00	0.00	0.00	1000	0.142	0.00	0.00	0.00	1008	0.126
		Sample Litter	**7.30**	6.97	7.60	1000	<0.001	**8.42**	8.13	8.72	1000	<0.001
		Sample Soil	**7.26**	6.94	7.55	1114	<0.001	**8.36**	8.08	8.63	1000	<0.001
	**Random**	Locality	0.08	0	0.2	1000	NA	0.06	0	0.2	521.6	NA
		Habitat	0.006	0	0.002	1000	NA	0	0	0.002	649.6	NA
**Birds versus Prokaryotes (16S)**	**Fix**	Richness taxa	0.00	0.00	0.00	1000.00	0.926	0.00	0.00	0.01	236.30	0.716
		Sample Litter	**5.04**	4.41	5.69	1105.00	<0.001	**8.30**	7.26	9.09	217.80	<0.001
		Sample Soil	**4.84**	4.15	5.46	1000.00	<0.001	**8.23**	7.13	8.96	385.80	<0.001
	**Random**	Locality	0.01	0	0.4	293.6	NA	0.1	0	0.3	1000	NA
		Habitat	0.3	0	0.9	1000	NA	0.1	0	0.5	1000	NA
**Trees versus Protists**	**Fix**	Richness taxa	0.00	0.00	0.00	1109.30	0.69	0.00	0.00	0.00	1000	0.92
		Marker 18S	**3.47**	3.11	3.81	944.40	<0.001	**4.58**	4.21	4.86	1122.00	<0.001
		Marker COI	**1.48**	1.13	1.85	1000.00	0.00	**3.59**	3.28	3.91	1449.00	<0.001
		Sample Litter	**2.09**	1.76	2.40	1000.00	<0.001	**2.19**	1.90	2.46	1000.00	<0.001
		Sample Soil	**1.98**	1.66	2.28	1000.00	<0.001	**2.04**	1.74	2.31	1000.00	<0.001
	**Random**	Locality	0.004	0	0.009	546.8	NA	0.004	0	0.01	174.3	NA
		Habita	0.02	0	0.0	1000	NA	0.009	0	0.02	1000	NA

		t			2							
**Birds versus Protists**	**Fix**	Richness taxa	0.00	0.00	0.00	1000.00	0.968	**0.00**	0.00	0.00	1000.00	0.96
		Marker 18S	**3.40**	2.92	4.01	1000.00	<0.001	**4.46**	4.02	4.90	1000.00	<0.001
		Marker COI	**1.52**	1.00	2.10	1000.00	<0.001	**3.56**	3.10	3.98	1000.00	<0.001
		Sample Litter	**2.27**	1.98	2.57	876.70	<0.001	**2.34**	2.07	2.56	1000.00	<0.001
		Sample Soil	**2.13**	1.85	2.40	1000.00	<0.001	**2.17**	1.93	2.41	905.00	<0.001
	**Random**	Locality	0.005	0	0.01	696.4	NA	0.003	0	0.01	811.9	NA
		Habitat	0.005	0	0.03	223.4	NA	0.006	0	0.02	653.4	NA
**Trees versus Fungi**	**Fix**	Richness taxa	0.00	0.00	0.00	1000.00	0.56	0.00	0.00	0.00	1000.00	0.82
		Marker 18S	**3.48**	3.20	3.81	1000.00	<0.001	**5.12**	4.68	5.59	1000.00	<0.001
		Marker COI	**3.03**	2.73	3.33	1084.20	<0.001	**4.44**	4.00	4.89	1122.00	<0.001
		Sample Litter	**2.08**	1.80	2.33	1000.00	<0.001	**1.22**	0.78	1.65	1000.00	<0.001
		Sample Soil	**1.97**	1.69	2.22	881.20	<0.001	**1.23**	0.79	1.62	1000.00	<0.001
	**Random**	Locality	0.008	0	0.03	886.8	NA	0.004	0	0.01	309.5	NA
		Habitat	0.002	0	0.005	599.6	NA	0.006	0	0.006	1000	NA
**Birds versus Fungi**	**Fixed**	Richness taxa	0.00	0.00	0.00	1182.00	0.88	0.00	0.00	0.00	1000.00	0.92
		Marker 18S	**3.47**	3.04	3.88	1000.00	<0.001	**5.10**	4.55	5.74	1000.00	<0.001
		Marker COI	**3.09**	2.67	3.54	1145.00	<0.001	**4.41**	3.76	5.01	1000.00	<0.001
		Sample Litter	**2.21**	1.95	2.45	1000.00	<0.001	**1.26**	0.90	1.61	1000.00	<0.001
		Sample Soil	**2.09**	1.85	2.30	1000.00	<0.001	**1.28**	0.91	1.64	1000.00	<0.001
	**Random**	Locality	0.002	0	0.002	806.6	NA	0.001	0	0.002	464.1	NA
		Habitat	0.006	0	0.02	1000	NA	0.004	0	0.009	466	NA
**Trees versus**	**Fix**	Richness taxa	0.00	0.00	0.00	1000.00	0.84	0.00	0.00	0.00	1000.00	0.93

**Metazoan**		Marker 18S	**3.32**	2.98	3.71	1000.00	<0.001	**4.78**	4.47	5.06	1040.00	<0.001
		Marker COI	**2.37**	2.01	2.74	1000.00	<0.001	**4.35**	4.04	4.63	1221.00	<0.001
		Sample Litter	**2.04**	1.69	2.33	1338.00	<0.001	**1.86**	1.61	2.14	1000.00	<0.001
		Sample Soil	**1.85**	1.54	2.18	1128.00	<0.001	**1.66**	1.43	1.95	1000.00	<0.001
	**Random**	Locality	0.005	0	0.02	131.9	NA	0.003	0	0.02	371.9	NA
		Habitat	0.007	0	0.02	1000	NA	0.008	0	0.003	606.8	NA
**Birds versus Metazoan**	**Fix**	Richness taxa	0.00	0.00	0.00	1000.00	0.83	0.00	0.00	0.00	647	0.80
		Marker 18S	**3.34**	2.77	3.80	1000.00	<0.001	**4.77**	4.25	5.19	1000.00	<0.001
		Marker COI	**2.49**	1.98	3.05	1000.00	<0.001	**4.43**	3.98	4.86	1000.00	<0.001
		Sample Litter	**2.13**	1.84	2.41	1000.00	<0.001	**1.97**	1.76	2.21	1000.00	<0.001
		Sample Soil	**1.95**	1.66	2.19	1000.00	<0.001	**1.75**	1.52	1.98	866.40	<0.001
	**Random**	Locality	0.003	0	0.01	224.6	NA	0.006	0	0.02	901.9	NA
		Habitat	0.01	0	0.04	1000	NA	0.007	0	0.03	455.9	NA

Table S4 - Bird species by locality and habitat. Localities are: BC = Bejamin Constant, JAU = National Park Jaú, CUI = Cuieras and CXN = National Forest Caxiuanã. The habitats are: CAM = campinas, IG = igapós, TF = terra-firmes and VZ = várzeas. See main text for a description of the localities and habitats sampled.

**Species**	**BC**	**JAU**	**CUI**	**CXN**
TINAMIDAE (10)				
Tinamus tao				TF
Tinamus major	TF	TF	TF	TF
Tinamus guttatus	TF	TF		TF
Crypturellus cinereus	TF	TF CAM		TF CAM
Crypturellus soui	TF	TF CAM	TF CAM	TF CAM
Crypturellus undulatus		IG		IG VZ
Cyrpturellus strigulosus				TF
Crypturellus variegatus	TF	TF	TF	TF
Crypturellus brevirostris			TF	
Crypturellus duidae		CAM		
CRACIDAE (13)				
Penelope marail			TF	
Penelope jacquacu		TF	TF	
Penelope pileata				TF
Aburria cumanensis		IG		
Aburria cujubi				IG VZ
Ortalis guttata	TF			
Ortalis motmot			TF CAM	

Nothocrax urumutum		TF IG		
Crax alector			TF	
Crax globulosa	VZ			
Crax fasciolata				TF
Pauxi tomentosa		IG	IG	
Pauxi tuberosa		IG CAM		
ODONTOPHORIDAE (1)				
Odontophorus gujanensis		TF	TF	TF
THRESKIORNITHIDAE (1)				
Mesembrinibis cayennensis	IG VZ	IG	IG	IG VZ
CATHARTIDAE (5)				
Cathartes aura	IG VZ	IG CAM	IG CAM	IG VZ CAM
Cathartes burrovianus	VZ			
Cathartes melambrotus	TF	TF	TF	TF
Coragyps atratus	IG VZ	IG CAM	IG CAM	IG VZ CAM
Sarcoramphus papa	TF IG VZ	TF IG	TF IG	TF IG VZ
ACCIPITRIDAE (26)				
Leptodon cayanensis	TF IG VZ	TF IG	TF IG	TF IG VZ
Chondrohierax uncinatus			TF	TF
Gampsonyx swainsonii		CAM	CAM	
Harpagus bidentatus	TF	TF	TF	TF
Accipiter poliogaster				TF IG VZ

Accipiter superciliosus			TF IG	
Accipiter bicolor		TF		TF
Busarellus nigricollis	IG VZ	IG CAM		IG VZ CAM
Rostrhamus sociabilis				VZ
Helicolestes hamatus	IG VZ			
Geranospiza caerulescens	IG VZ	IG	IG	IG VZ
Buteogallus schistaceus		IG		IG VZ
Buteogallus meridionalis			CAM	
Urubutinga urubitinga				
Rupornis magnirostris	IG VZ	IG CAM	IG CAM	IG VZ CAM
Geranoaetus albicaudatus				
Pseudastur albicollis			TF	TF
Leucopternis melanops		TF	TF	
Leucopternis kuhli				TF
Buteo nitidus		TF IG CAM	TF IG CAM	TF IG VZ CAM
Buteo brachyurus	TF IG VZ	TF IG CAM	TF IG CAM	TF IG VZ CAM
Morphnus guianensis			TF	TF
Harpia harpyja	TF	TF	TF	TF
Spizaetus tyrannus	TF IG VZ	TF IG	TF IG	TF IG VZ
Spizaetus melanoleucus				TF IG VZ
Spizaetus ornatus	TF	TF	TF	TF
PSOPHIIDAE (3)				
Psophia crepitans		TF	TF	
Psophia leucoptera	TF			
Psophia viridis				TF

COLUMBIDAE (9)				
Columbina passerina		CAM	CAM	
Columbina talpacoti	VZ	CAM	CAM	VZ CAM
Patagioenas speciosa		TF IG CAM	TF IG CAM	TF IG VZ CAM
Patagioenas cayennensis	IG VZ	IG CAM	IG CAM	IG VZ CAM
Patagioenas plumbea	TF IG VZ	TF IG CAM	TF IG CAM	TF IG VZ CAM
Patagioenas subvinacea	TF IG VZ	TF IG CAM	TF IG CAM	TF IG VZ CAM
Leptotila verreauxi	IG VZ	IG CAM	IG CAM	IG VZ CAM
Leptotila rufaxilla	TF IG VZ	TF IG CAM	TF IG CAM	TF IG VZ CAM
Geotrygon montana	TF	TF CAM	TF CAM	TF CAM
OPISTHOCOMIDAE (1)				
Opisthocomus hoazin	VZ			VZ
CUCULIDAE (9)				
Coccycua minuta	VZ			VZ
Piaya cayana	IG VZ	IG CAM	IG CAM	IG VZ CAM
Piaya melanogaster	TF	TF	TF	TF
Coccyzus melacoryphus	IG CAM	IG CAM	IG CAM	
Crotophaga major	IG VZ	IG	IG	IG VZ
Crotophaga ani	IG VZ	IG CAM	IG CAM	IG VZ CAM
Tapera naevia	VZ	CAM		
Dromococcyx pavoninus		TF	TF	
Neomorphus squamiger				TF

STRIGIDAE (10)				
Megascops choliba	IG VZ	IG CAM	IG CAM	IG VZ CAM
Megascops watsonii			TF	
Megascops usta	TF	TF		TF
Lophostrix cristata		TF	TF	TF
Pulsatrix perspicillata	TF IG VZ	TF IG CAM	TF IG CAM	TF IG VZ CAM
Strix virgata			TF	TF
Strix huhula		TF	TF	TF
Glaucidium hardyi			TF	TF
Glaucidium brasilianum		CAM		
Asio stygius		CAM		CAM
NYCTIBIIDAE (5)				
Nyctibius grandis	IG VZ	IG CAM	IG CAM	IG VZ CAM
Nyctibius aethereus		TF	TF	TF
Nyctibius griseus	TF IG VZ	TF IG CAM	TF IG CAM	TF IG VZ CAM
Nyctibius leucopterus		TF CAM	TF CAM	TF CAM
Nyctibius bracteatus		TF	TF	
CAPRIMULGIDAE (12)				
Nyctiphrynus ocellatus				TF
Antrostomus rufus		CAM		
Lurocalis semitorquatus	IG VZ	IG CAM	IG CAM	IG VZ CAM
Nyctiprogne leucopyga	IG VZ	IG	IG	
Nyctidromus nigrescens	TF	TF	TF	TF
Nyctidromus albicollis	IG VZ	IG CAM	IG CAM	IG VZ CAM

Caprimulgus maculicaudus			CAM	
Hydropsalis cayennensis		CAM		
Hydropsalis climacocerca	VZ			
Nannochordeiles pusillus		CAM	CAM	
Chordeiles rupestris	VZ			
Chordeiles acutipennis		CAM	CAM	
APODIDAE (9)				
Streptoprocne zonaris			TF	
Chaetura spinicaudus	TF	TF	TF	TF
Chaetura cinereiventris	TF IG VZ	TF IG CAM	TF IG CAM	TF IG VZ CAM
Chaetura chapmani		TF	TF	
Chaetura viridipennis	TF		TF	
Chaetura meridionalis			IG CAM	
Chaetura brachyura	TF IG VZ	TF IG CAM	TF IG CAM	TF IG VZ CAM
Tachornis squamata	TF IG VZ	TF IG CAM	TF IG CAM	TF IG VZ CAM
Panyptila cayennensis		CAM		
TROCHILIDAE (30)				
Glaucis hirsutus	TF IG VZ	TF IG CAM		TF IG VZ CAM
Threnetes leucurus	TF IG VZ	TF IG		TF IG VZ
Phaethornis rupurumii		TF IG CAM		
Phaethornis ruber	TF	TF CAM	TF CAM	TF CAM
Phaethornis philippii	TF			
Phaethornis bourcieri		TF	TF	TF
Phaethornis superciliosus			TF	TF

Phaethornis malaris	TF	TF		
Campylopterus largipennis		TF CAM	TF CAM	TF CAM
Florisuga mellivora	TF	TF	TF	TF
Anthracothorax nigricollis	VZ	CAM	CAM	VZ CAM
Avocettula recurvirostris			TF CAM	TF CAM
Topaza pella			IG CAM	IG CAM
Topaza pyra		IG CAM		
Chrysolampis mosquitus		TF		
Discosura langsdorffi	TF			
Discosura longicaudus			TF CAM	TF CAM
Chlorestes notata				IG VZ CAM
Chlorostilbon mellisugus	IG VZ	IG CAM		
Chlorestes notata	IG VZ	IG CAM	IG CAM	
Thalurania furcata	TF	TF	TF	TF
Hylocharis sapphirina		TF CAM	TF CAM	TF CAM
Hylocharis cyanus	TF	TF CAM	TF CAM	TF CAM
Polytmus theresiae		CAM	CAM	CAM
Amazilia versicolor		CAM	CAM	CAM
Amazilia fimbriata	IG VZ	IG CAM	IG CAM	IG VZ CAM
Heliodoxa aurescens	TF	TF		TF
Heliothryx auritus	TF	TF	TF	TF
Heliomaster longirostris	VZ			VZ
TROGONIDAE (8)				
Trogon melanurus	TF	TF	TF	TF
Trogon viridis	TF IG VZ	TF IG CAM	TF IG CAM	TF IG VZ CAM

Trogon ramonianus	TF		TF	
Trogon violaceus		TF IG		TF IG VZ
Trogon curucui	TF IG VZ	TF IG CAM	TF IG CAM	
Trogon rufus	TF	TF	TF	TF
Trogon collaris	TF			
Pharomachrus pavoninus	TF	TF	TF	
ALCEDINIDAE (2)				
Chloroceryle aenea	TF IG VZ	TF IG	TF IG	TF IG VZ
Chloroceryle inda	TF IG VZ	TF IG	TF IG	TF IG VZ
MOMOTIDAE (3)				
Electron platyrhynchum	TF			
Baryphthengus martii	TF			
Momotus momota	TF IG VZ	TF IG	TF IG	TF IG VZ
GALBULIDAE (9)				
Galbula albirostris		TF	TF	
Galbula cyanicollis	TF			TF
Galbula ruficauda				VZ CAM
Galbula galbula		IG CAM		
Galbula cyanescens	VZ			
Galbula chalcothorax	TF			
Galbula leucogastra		CAM	CAM	
Galbula dea	TF	TF	TF	TF
Jacamerops aureus	TF	TF	TF	TF

BUCCONIDAE (17)				
Notharchus hyperrhynchus				TF
Notharchus macrorhynchos		TF	TF	
Notharchus ordii	TF	TF CAM		
Notharchus tectus	TF IG VZ	TF IG CAM	TF IG CAM	TF IG VZ CAM
Bucco macrodactylus		TF IG		
Bucco tamatia		TF IG CAM	TF IG CAM	TF IG VZ CAM
Bucco capensis		TF	TF	TF
Malacoptila fusca		TF	TF	
Malacoptila rufa	TF			TF
Micromonacha lanceolata		TF		
Nonnula rubecula	TF	TF	TF	TF
Nonnula ruficapilla	TF			TF
Nonnula amaurocephala		IG		
Monasa atra			TF	
Monasa nigrifrons	IG VZ	IG	IG	
Monasa morphoeus	TF	TF		TF
Chelidoptera tenebrosa	IG VZ	IG CAM	IG CAM	IG VZ CAM
CAPITONIDAE (4)				
Capito aurovirens	VZ			
Capito niger			TF	
Capito auratus	TF IG VZ	TF IG		
Eubucco richardsoni	VZ			

RAMPHASTIDAE (14)				
Ramphastos toco				VZ
Ramphastos tucanus	TF IG VZ	TF IG CAM	TF IG CAM	TF IG VZ CAM
Ramphastos vitellinus	TF IG VZ	TF IG CAM	TF IG CAM	TF IG VZ CAM
Selenidera piperivora	TF		TF	
Selenidera reinwardtii	TF			
Selenidera nattereri		TF		
Selenidera gouldii				TF
Pteroglossus viridis			TF	
Pteroglossus inscriptus	TF		TF	TF
Pteroglossus bitorquatus				TF IG VZ
Pteroglossus azara	IG VZ	IG		
Pteroglossus aracari		TF IG CAM	TF IG CAM	TF IG VZ CAM
Pteroglossus castanotis	IG VZ	IG		
Pteroglossus pluricinctus	TF	TF		
PICIDAE (20)				
Picumnus aurifrons	TF			
Picumnus lafresnayi		IG		
Picumnus exilis			TF	
Picumnus castelnau	VZ			
Melanerpes cruentatus	IG VZ	IG	IG	IG VZ
Veniliornis cassini		TF IG	TF IG	
Veniliornis affinis	TF	TF		TF
Veniliornis passerinus	VZ		CAM	VZ CAM
Piculus leucolaemus				VZ

Piculus flavigula	TF	TF	TF	TF
Piculus chrysochlorus		TF	TF	TF
Celeus torquatus	TF IG VZ	TF IG	TF IG	TF IG VZ
Celeus elegans	TF IG VZ	TF IG	TF IG	TF IG VZ
Celeus undatus			TF	TF
Celeus grammicus	TF	TF	TF	
Celeus flavus	IG VZ	IG CAM	IG CAM	IG VZ CAM
Colaptes punctigula	VZ			
Dryocopus lineatus	IG VZ	IG CAM	IG CAM	IG VZ CAM
Campephilus rubricollis	TF	TF	TF	TF
Campephilus melanoleucos	TF IG VZ	TF IG	TF IG	
FALCONIDAE (12)				
Daptrius ater	TF IG VZ	TF IG CAM	TF IG CAM	TF IG VZ CAM
Ibycter americanus	TF IG VZ	TF IG CAM	TF IG CAM	TF IG VZ CAM
Caracara cheriway			CAM	
Caracara plancus			CAM	
Milvago chimachima	IG VZ	IG CAM	IG CAM	
Herpetotheres cachinnans	VZ	CAM		VZ CAM
Micrastur ruficollis	TF IG VZ	TF IG	TF IG	TF IG VZ
Micrastur gilvicollis	TF	TF	TF	
Micrastur mintoni				TF
Micrastur mirandollei	TF	TF	TF	TF
Micrastur semitorquatus	TF IG VZ	TF IG	TF IG	TF IG VZ
Falco rufigularis	TF IG VZ	TF IG CAM	TF IG CAM	TF IG VZ CAM

PSITTACIDAE (35)

Ara ararauna	TF IG VZ	TF IG CAM	TF IG CAM	TF IG VZ CAM
Ara macao	TF IG VZ	TF IG CAM	TF IG CAM	TF IG VZ CAM
Ara chloropterus	TF IG VZ	TF IG CAM	TF IG CAM	TF IG VZ CAM
Ara severus	VZ			VZ
Orthopsittaca manilata	VZ	CAM	CAM	VZ CAM
Guarouba guarouba				TF IG
Psittacara leucophthalmus	VZ	CAM	CAM	
Eupsittula pertinax		IG CAM		
Aratinga weddellii	VZ			
Pyrrhura picta	TF IG VZ		TF IG	
Pyrrhura melanura		TF		
Pyrrhura perlata				TF
Forpus xanthopterygius	VZ			
Forpus modestus	VZ			
Brotogeris versicolurus	VZ			VZ
Brotogeris cyanoptera	TF IG			
Brotogeris chrysoptera		TF IG	TF IG	TF IG VZ
Brotogeris sanctithomae	VZ			
Touit huetii		TF CAM	TF CAM	TF CAM
Touit purpuratus	TF	TF CAM	TF CAM	TF CAM
Pionites melanocephalus		TF	TF	
Pionites leucogaster	TF			TF
Pyrilia barrabandi	TF	TF		
Pyrilia caica			TF	
Pionus menstruus	TF IG VZ	TF IG	TF IG	TF IG VZ

Pionus fuscus		TF	TF	TF
Amazona festiva	VZ			
Amazona kawalli	IG VZ	IG		
Amazona farinosa	TF	TF	TF	TF
Amazona amazonica	TF IG VZ	TF IG CAM	TF IG CAM	TF IG VZ CAM
Amazona autumnalis		TF	TF	
Amazona ochrocephala			IG CAM	
Deroptyus accipitrinus		TF	TF	TF
Graydidascalus brachyurus	VZ			
Gypopsitta vulturina				TF
THAMNOPHILIDAE (90)				
Euchrepomis humeralis	TF			
Euchrepomis spodioptila		TF	TF	
Myrmornis torquata			TF	TF
Pygiptila stellaris	TF IG VZ	TF IG		TF IG VZ
Microrhopias quixensis	TF IG VZ	TF IG CAM	TF IG CAM	
Neoctantes niger	TF			
Epinecrophylla gutturalis			TF	
Epinecrophylla leucophthalma				TF
Epinecrophylla haematonota	TF	TF		
Epinecrophylla ornata	TF			
Epinecrophylla erythrura	TF			
Aprositornis disjuncta		CAM		
Myrmophylax atrothorax	TF IG	TF IG	TF IG	TF IG
Myrmotherula brachyura	TF IG VZ	TF IG CAM	TF IG CAM	TF IG VZ CAM

Myrmotherula obscura		TF		
Myrmotherula ambigua		TF		
Myrmotherula sclateri	TF			
Myrmotherula multostriata	IG	IG		IG
Myrmotherula cherriei		IG CAM		
Myrmotherula klagesi		IG		
Myrmotherula axillaris	TF IG VZ	TF IG CAM	TF IG CAM	TF IG VZ CAM
Myrmotherula longipennis	TF	TF	TF	TF
Myrmotherula menetriesii	TF	TF	TF	TF
Myrmotherula assimilis	IG VZ	IG		
Formicivora grisea		CAM	CAM	CAM
Isleria hauxwelli	TF			TF
Isleria guttata			TF	
Thamnomanes ardesiacus	TF	TF	TF	
Thamnomanes saturninus	TF			
Thamnomanes caesius	TF IG VZ	TF IG	TF IG	TF IG VZ
Thamnomanes schistogynus	TF			
Dichrozona cincta		TF		TF
Megastictus margaritatus		TF		
Herpsilochmus dorsimaculatus		TF IG CAM	TF IG CAM	
Herpsilochmus rufimarginatus				TF IG VZ
Sakesphorus canadensis		IG CAM	IG CAM	
Sakesphorus luctuosus				VZ
Thamnophilus doliatus	VZ		CAM	
Thamnophilus schistaceus	TF	TF	TF	TF
Thamnophilus murinus	TF	TF	TF	

Thamnophilus cryptoleucus	VZ			
Thamnophilus nigrocinereus		IG		
Thamnophilus punctatus			TF IG	
Thamnophilus stictocephalus				TF CAM
Thamnophilus aethiops	TF	TF		TF
Thamnophilus amazonicus	TF IG	TF IG	TF IG	
Cymbilaimus lineatus	TF	TF	TF	TF
Taraba major	VZ	CAM		VZ CAM
Frederickena viridis			TF	
Frederickena unduligera	TF	TF		
Myrmoderus ferrugineus			TF	
Hypocnemoides maculicauda	IG VZ			
Hypocnemoides melanopogon	IG VZ	IG		IG VZ
Hylophylax naevius	TF IG	TF IG	TF IG	TF IG
Hylophylax punctulatus	IG VZ	IG		IG VZ
Sclateria naevia		IG		IG VZ
Myrmelastes leucostigma	TF	TF	TF	TF
Myrmelastes schistaceus	TF			
Myrmelastes hyperythrus	TF			
Myrmoborus melanurus	VZ			
Myrmoborus lophotes				
Myrmoborus myotherinus	TF IG	TF IG		TF IG
Myrmoborus leucophrys	TF IG VZ			
Myrmoborus lugubris		IG	IG	
Pyriglena leuconota				TF
Percnostola rufifrons		TF CAM	TF CAM	

Percnostola minor		TF CAM		
Akletos melanoceps	VZ			
Hafferia fortis	TF			
Sciaphylax hemimelaena	TF			
Cercomacra cinerascens	TF IG VZ	TF IG	TF IG	TF IG VZ
Cercomacroides nigrescens	TF		TF	
Cercomacroides fuscicauda	TF			
Cercomacroides laeta			TF CAM	
Cercomacroides tyrannina		TF IG	TF IG	
Cercomacroides serva	TF			
Hypocnemis hypoxantha	TF	TF		
Hypocnemis cantator	TF IG VZ	TF IG CAM	TF IG CAM	TF IG VZ CAM
Hypocnemis flavescens		TF		
Hypocnemis peruviana	TF			
Pithys albifrons		TF CAM	TF CAM	
Willisornis poecilinotus	TF	TF	TF	TF
Phlegopsis nigromaculata	TF			TF
Phlegopsis erythroptera	TF	TF	TF	
Gymnopithys leucaspis		TF		
Gymnopithys rufigula			TF	
Oneillornis salvini	TF			
Rhegmatorhina cristata		TF		
Rhegmatorhina melanosticta	TF			
CONOPOPHAGIDAE (1)				
Conopophaga aurita	TF	TF	TF	TF

GRALLARIDAE (3)				
Grallaria varia		TF	TF	TF
Hylopezus macularius		TF	TF	TF
Myrmothera campanisona	TF	TF	TF	
RHINOCRYPTIDAE (1)				
Liosceles thoracicus	TF			
FORMICARIIDAE (2)				
Formicarius colma	TF	TF	TF	TF
Formicarius analis	TF	TF	TF	TF
SCLERURIDAE (3)				
Sclerurus rufigularis	TF	TF	TF	TF
Sclerurus caudacutus		TF	TF	TF
Sclerurus macconnelli			TF	TF
DENDROCOLAPTIDAE (27)				
Dendrocincla fuliginosa	TF	TF	TF	TF
Dendrocincla merula		TF	TF	TF
Deconychura longicauda	TF	TF	TF	TF
Certhiasomus stictolaemus	TF	TF	TF	TF
Sittasomus griseicapillus	TF	TF	TF	TF
Glyphorynchus spirurus	TF	TF	TF	TF
Xiphorhynchus pardalotus			TF	

Xiphorhynchus ocellatus	TF	TF		
Xiphorhynchus elegans	TF			
Xiphorhynchus spixii				TF
Xiphorhynchus obsoletus	IG VZ	IG CAM	IG CAM	IG VZ CAM
Xiphorhynchus guttatus	TF IG VZ	TF IG CAM	TF IG CAM	TF IG VZ CAM
Campylorhamphus procurvoides		TF	TF	TF
Dendroplex picus	IG VZ	IG CAM	IG CAM	IG VZ CAM
Dendroplex kienerii	VZ			
Lepidocolaptes albolineatus			TF	
Lepidocolaptes duidae		TF		
Lepidocolaptes lyardi				TF
Nasica longirostris	IG VZ	IG		IG VZ
Dendrexetastes rufigula	TF	TF	TF	TF
Dendrocolaptes certhia	TF	TF CAM	TF CAM	TF CAM
Dendrocolaptes picumnus	TF	TF	TF	
Xiphocolaptes promeropirhynchus	TF	TF		
Xiphocolaptes carajaensis				TF
Hylexetastes stresemanni		TF		
Hylexetastes perrotii			TF	
Hylexetastes brigidai				TF
XENOPIDAE (3)				
Xenops tenuirostris	TF		TF	
Xenops minutus	TF	TF	TF	TF
Xenops rutilans			TF	

FURNARIIDAE (19)				
Berlepschia rikeri	1	1	1	
Microxenops milleri	TF	TF	TF	
Furnarius leucopus				
Ancistrops strigilatus	TF	TF		
Clibanornis obscurus		TF	TF	
Automolus rufipileatus	VZ			VZ
Automolus subulatus	TF	TF		
Automolus ochrolaemus	TF	TF	TF	
Automolus infuscatus	TF	TF	TF	
Automolus paraensis				TF
Anabacerthia ruficaudata	TF			TF
Philydor erythrocercum	TF		TF	TF
Philydor erythropterum	TF			TF
Philydor pyrrhodes	TF	TF	TF	TF
Synallaxis rutilans		TF	TF	TF
Synallaxis gujanensis				
Metopothrix aurantiaca	VZ			
Cranioleuca vulpina	VZ			
Cranioleuca gutturata	IG VZ	IG		
PIPRIDAE (17)				
Neopelma chrysocephalum		CAM	CAM	
Tyranneutes stolzmanni	TF	TF	TF	TF
Tyranneutes virescens			TF	
Pipra filicauda	VZ			

Pipra fasciicauda	VZ			VZ
Ceratopipra erythrocephala		TF	TF	
Ceratopipra rubrocapilla	TF			TF
Lepidothrix coronata	TF	TF		
Lepidothrix serena			TF	
Manacus manacus	TF		TF CAM	TF CAM
Heterocercus flavivertex		IG	IG	
Heterocercus linteatus				IG CAM
Machaeropterus regulus	TF			
Dixiphia pipra	TF IG VZ	TF IG CAM	TF IG CAM	TF IG VZ CAM
Xenopipo atronitens		CAM	CAM	
Corapipo gutturalis			TF	
Chiroxiphia pareola	TF	TF		
OXYRUNCIDAE (3)				
Myiobius atricaudus			TF	
Myiobius barbatus	TF IG VZ	TF IG	TF IG	TF IG VZ
Terenotriccus erythrurus	TF	TF	TF	TF
ONYCHORHYNCHIDAE (1)				
Onychorhynchus coronatus	TF	TF	TF	TF
TITYRIDAE (14)				
Schiffornis major	VZ			
Schiffornis turdina	TF	TF	TF	TF
Laniocera hypopyrra	TF	TF	TF	TF

Iodopleura isabellae	TF	TF		TF
Iodopleura fusca			TF	
Tityra inquisitor		TF IG CAM		TF IG VZ CAM
Tityra cayana		TF IG CAM		TF IG VZ CAM
Tityra semifasciata				VZ
Pachyramphus rufus	VZ			
Pachyramphus castaneus	VZ			
Pachyramphus polychopterus	TF IG VZ	TF IG	TF IG	TF IG VZ
Pachyramphus marginatus	TF IG VZ	TF IG	TF IG	TF IG VZ
Pachyramphus surinamus		TF	TF	
Pachyramphus minor			TF IG	TF IG VZ
COTINGIDAE (15)				
Rupicola rupicola			TF	
Phoenicircus carnifex			TF	TF
Phoenicircus nigricollis		TF		
Haematoderus militaris			TF	
Querula purpurata	TF IG VZ		TF IG	TF IG VZ
Perissocephalus tricolor		TF		
Cephalopterus ornatus	VZ			VZ
Lipaugus vociferans	TF IG VZ	TF IG	TF IG	TF IG VZ
Cotinga maynana	VZ			
Cotinga cayana	TF IG VZ	TF IG	TF IG	TF IG VZ
Cotinga cotinga			TF	TF
Porphyrolaema porphyrolaema	TF			
Gymnoderus foetidus	VZ			VZ

Xipholena punicea	TF	TF	TF	
Xipholena lamellipennis			TF	TF
PIPRITIDAE (1)				
Piprites chloris	TF	TF	TF	TF
PLATYRICHIDAE (4)				
Neopipo cinnamomea		TF	TF	
Platyrinchus saturatus	TF		TF	TF
Platyrinchus coronatus	TF	TF	TF	
Platyrinchus platyrhynchos	TF	TF	TF	TF
RHYNCHOCYCLIDAE (24)				
Cnipodectes subbrunneus	TF	TF		
Mionectes oleagineus	TF	TF	TF	TF
Mionectes macconnelli		TF	TF	TF
Corythopis torquatus	TF	TF	TF	TF
Phylloscartes virescens			TF	
Rhynchocyclus olivaceus	TF		TF	TF
Tolmomyias sulphurescens	VZ			VZ
Tolmomyias assimilis	TF	TF	TF	TF
Tolmomyias poliocephalus	TF IG VZ	TF IG CAM	TF IG CAM	
Tolmomyias flaviventris	IG VZ			
Todirostrum maculatum	IG VZ	IG CAM	IG CAM	
Todirostrum pictum		TF	TF	
Todirostrum chrysocrotaphum	TF	TF	TF	TF

Poecilotriccus latirostris	VZ			
Myiornis ecaudatus	TF IG VZ	TF IG	TF IG	TF IG VZ
Hemitriccus minor	IG VZ	IG	IG	IG VZ
Hemitriccus josephinae			TF	
Hemitriccus zosterops	TF	TF	TF	
Hemitriccus griseipectus				TF
Hemitriccus iohannis	TF			
Hemitriccus inornatus			CAM	
Hemitriccus minimus		CAM		CAM
Lophotriccus vitiosus	TF	TF	TF	
Lophotriccus galeatus	TF IG VZ	TF IG CAM	TF IG CAM	
TYRANNIDAE (56)				
Zimmerius acer			TF IG	
Zimmerius gracilipes	TF IG VZ	TF IG CAM		TF IG VZ CAM
Stigmatura napensis	VZ			
Inezia subflava		IG		
Ornithion inerme	TF IG	TF IG	TF IG	TF IG
Camptostoma obsoletum	VZ	CAM	CAM	VZ CAM
Elaenia flavogaster				VZ CAM
Elaenia spectabilis	VZ			
Elaenia parvirostris	TF IG VZ	TF IG CAM		
Elaenia pelzelni	VZ			
Elaenia cristata		CAM		
Elaenia ruficeps		CAM	CAM	
Myiopagis gaimardii	TF IG VZ	TF IG CAM	TF IG CAM	TF IG VZ CAM

Myiopagis caniceps	TF	TF	TF	TF
Myiopagis flavivertex	VZ			
Myiopagis viridicata				TF CAM
Tyrannulus elatus	TF IG VZ	TF IG CAM	TF IG CAM	TF IG VZ CAM
Phaeomyias murina		IG CAM	IG CAM	IG VZ CAM
Serpophaga hypoleuca	VZ			
Atilla cinnamomeus				IG VZ
Attila citriniventris		CAM		
Atilla spadiceus	TF	TF	TF	TF
Legatus leucophaius	IG VZ	IG CAM	IG CAM	IG VZ CAM
Ramphotrigon ruficauda	TF	TF	TF	TF
Ramphotrigon fuscicauda	TF			
Myiarchus tuberculifer	IG VZ	IG CAM	IG CAM	IG VZ CAM
Myiarchus swainsoni	VZ	CAM	CAM	
Myiarchus ferox	IG VZ	IG CAM	IG CAM	IG VZ CAM
Myiarchus tyrannulus			IG CAM	
Sirystes albocinereus	TF			
Sirystes subcanescens			TF	
Sirystes sibilator			TF	TF
Rhytipterna simplex	TF IG VZ	TF IG	TF IG	TF IG VZ
Rhytipterna immunda		CAM	CAM	
Pitangus sulphuratus	IG VZ	IG CAM	IG CAM	IG VZ CAM
Philohydor lictor	IG VZ	IG	IG	IG VZ
Myiodynastes maculatus	VZ	CAM	CAM	VZ CAM
Tyrannopsis sulphurea	VZ			VZ
Megarynchus pitangua	IG VZ	IG CAM	IG CAM	IG VZ CAM

Myiozetetes cayanensis	IG VZ	IG CAM	IG CAM	IG VZ CAM
Myiozetetes similis	IG VZ	IG CAM	IG CAM	IG VZ CAM
Myiozetetes luteiventris	VZ			
Tyrannus melancholicus	IG VZ	IG CAM	IG CAM	IG VZ CAM
Tyrannus savana	VZ	CAM	CAM	VZ CAM
Empidonomus varius		IG CAM	IG CAM	IG VZ CAM
Griseotyrannus aurantioatrocristatus	VZ		CAM	
Conopias trivirgatus				VZ
Conopias parvus	TF	TF IG CAM	TF IG CAM	
Colonia colonus				TF
Myiophobus fasciatus			IG	
Sublegatus obscurior		TF IG CAM		
Pyrocephalus rubinus	VZ			
Ochthornis littoralis	VZ			
Cnemotriccus fuscatus	VZ	CAM	CAM	VZ CAM
Lathrotriccus euleri		IG CAM		
Knipolegus poecilocercus	IG VZ	IG		
VIREONIDAE (8)				
Cyclarhis gujanensis		TF IG CAM	TF IG CAM	TF IG VZ CAM
Vireolanius leucotis	TF	TF	TF	TF
Hylophilus semicinereus	IG	IG CAM	IG CAM	IG CAM
Hylophilus brunneiceps		IG CAM		
Hylophilus thoracicus	TF	TF	TF	
Tunchiornis ochraceiceps	TF	TF	TF	TF
Pachysylvia hypoxantha	TF	TF		TF

Pachysylvia muscicapinus			TF	
CORVIDAE (2)				
Cyanocorax violaceus	IG VZ			
Cyanocorax cayanus			TF	
HIRUNDINIDAE (4)				
Stelgidopteryx ruficollis	VZ	CAM	CAM	
Progne chalybea	VZ			VZ
Hirundo rustica	VZ			
Atticora tibialis			TF CAM	
TROGLODYTIDAE (9)				
Microcerculus marginatus	TF			TF
Microcerculus bambla		TF	TF	
Troglodytes musculus	VZ	CAM	CAM	VZ CAM
Campylorhynchus turdinus	VZ			
Pheugopedius genibarbis	TF			
Pheugopedius coraya		TF	TF	TF
Cantorchilus leucotis	IG VZ	IG	IG	IG VZ
Cantorchilus griseus	VZ			
Cyphorhinus arada		TF	TF	
DONACOBIIDAE (1)				
Donacobius atricapilla	VZ			

POLIOPTILIDAE (5)				
Microbates collaris	TF	TF	TF	
Ramphocaenus melanurus	TF IG VZ	TF IG CAM	TF IG CAM	TF IG VZ CAM
Polioptila plumbea	IG VZ	IG CAM		IG VZ CAM
Polioptila guianensis			TF	
Polioptila facilis		TF		
TURDIDAE (7)				
Turdus leucomelas	IG VZ	IG CAM	IG CAM	IG VZ CAM
Turdus fumigatus	VZ	CAM	CAM	VZ CAM
Turdus hauxwelli	TF	TF		
Turdus sanchezorum	VZ			
Turdus lawrencii	TF			
Turdus ignobilis	TF		TF CAM	
Turdus albicollis		TF	TF	TF
PASSERELLIDAE (2)				
Ammodramus aurifrons	VZ	CAM	CAM	
Arremon taciturnus		TF	TF	TF
PARULIDAE (2)				
Myiothlypis rivularis			1	
Myiothlypis fulvicauda			TF	TF
ICTERIDAE (16)				
Psarocolius angustifrons	VZ			

Psarocolius viridis	TF	TF	TF	TF
Psarocolius decumanus	TF	TF	TF	
Psarocolius bifasciatus	VZ			VZ
Cacicus haemorrhous		TF	TF	
Cacicus latirostris	VZ			
Cacicus cela	IG VZ	IG CAM	IG CAM	IG VZ CAM
Icterus cayanensis	IG VZ		IG CAM	IG VZ CAM
Icterus chrysocephalus		CAM		
Icterus croconotus	VZ			
Gymnomystax mexicanus				
Lampropsar tanagrinus	VZ			
Chrysomus icterocephalus	VZ			
Molothrus oryzivorus	IG VZ	IG CAM	IG CAM	IG VZ CAM
Molothrus bonariensis	VZ		CAM	VZ CAM
Sturnella militaris		CAM	CAM	
MITROSPINGIDAE (1)				
Lamprospiza melanoleuca			TF	TF
THRAUPIDAE (57)				
Cissopis leverianus	TF	TF		
Schistochlamys melanopis		CAM		
Paroaria gularis	IG VZ	IG	IG	IG VZ
Tangara gyrola			TF	TF
Tangara schrankii	TF			
Tangara mexicana	TF IG VZ	TF IG CAM	TF IG CAM	TF IG VZ CAM

Tangara chilensis	TF	TF	TF	TF
Tangara velia	TF	TF	TF	TF
Tangara varia			TF	
Tangara punctata		TF	TF	TF
Tangara xanthogastra	TF			
Tangara episcopus	IG VZ	IG CAM	IG CAM	IG VZ CAM
Tangara palmarum	IG VZ	IG CAM	IG CAM	IG VZ CAM
Tangara cayana		CAM	CAM	
Nemosia pileata	VZ			
Cyanicterus cyanicterus			TF CAM	TF CAM
Conirostrum speciosum			IG	
Sicalis flaveola			CAM	
Chlorophanes spiza	TF IG VZ	TF IG CAM	TF IG CAM	TF IG VZ CAM
Hemithraupis flavicollis	TF	TF	TF	
Hemithraupis guira		TF IG		TF IG VZ
Volatinia jacarina	VZ	CAM	CAM	VZ CAM
Eucometis penicillata	IG VZ	IG		IG VZ
Lanio versicolor	TF			TF
Lanio fulvus		TF	TF	
*Maschalethraupis surinama**	TF	TF	TF	TF
*Islerothraupis luctuosa**	VZ			VZ
*Islerothraupis cristata**		TF CAM	TF CAM	TF CAM
*Islerothraupis rufiventer**	TF		TF	
Tachyphonus phoenicius		CAM	CAM	
Tachyphonus rufus			CAM	CAM
Ramphocelus nigrogularis	VZ			

Ramphocelus carbo	IG VZ	IG CAM	IG CAM	IG VZ CAM
Tersina viridis	TF	TF CAM	TF CAM	
Cyanerpes nitidus	TF	TF	TF	
Cyanerpes caeruleus	TF	TF CAM	TF CAM	TF CAM
Cyanerpes cyaneus	TF	TF	TF	TF
Dacnis cayana		TF IG CAM	TF IG CAM	TF IG VZ CAM
Dacnis flaviventer	VZ			
Dacnis lineata	TF		TF CAM	TF CAM
Coereba flaveola	IG VZ	IG CAM	IG CAM	IG VZ CAM
Sporophila lineola				VZ
Sporophila schistacea				
Sporophila plumbea				
Sporophila americana				VZ
Sporophila murallae	VZ			
Sporophila bouvronides	VZ			
Sporophila nigricollis		CAM		
Sporophila minuta				
Sporophila castaneiventris	VZ			
Sporophila angolensis	VZ	CAM	CAM	VZ CAM
Dolospingus fringilloides		CAM	CAM	
Emberizoides herbicola		CAM		
Saltator maximus	TF	TF	TF	TF
Saltator coerulescens	VZ		CAM	VZ CAM
Saltator grossus	TF	TF	TF	TF

CARDINALIDAE (5)

Habia rubica		TF		TF
Granatellus pelzelni				IG CAM
Caryothraustes canadensis	TF	TF	TF	
Periporphyrus erythromelas				TF
Cyanoloxia rothschildii	TF			TF
FRINGILLIDAE (9)				
Euphonia plumbea		CAM	CAM	
Euphonia chlorotica	TF IG VZ	TF IG CAM	TF IG CAM	TF IG VZ CAM
Euphonia violacea	IG VZ			IG VZ CAM
Euphonia laniirostris	VZ			
Euphonia chrysopasta	TF IG VZ	TF IG	TF IG	
Euphonia minuta	TF	TF	TF	
Euphonia xanthogaster	TF			
Euphonia rufiventris	TF IG VZ	TF IG		
Euphonia cayennensis			TF	TF

*Included within *Lanio* by CBRO, but we follow Burns et al. (2016)

Burns, K.J., Unitt, P. and Mason, N.A. 2016. A genus-level classification of the family Thraupidae (Class Aves: Order Passeriformes). Zootaxa, 4088(3), pp.329-354

Figures:

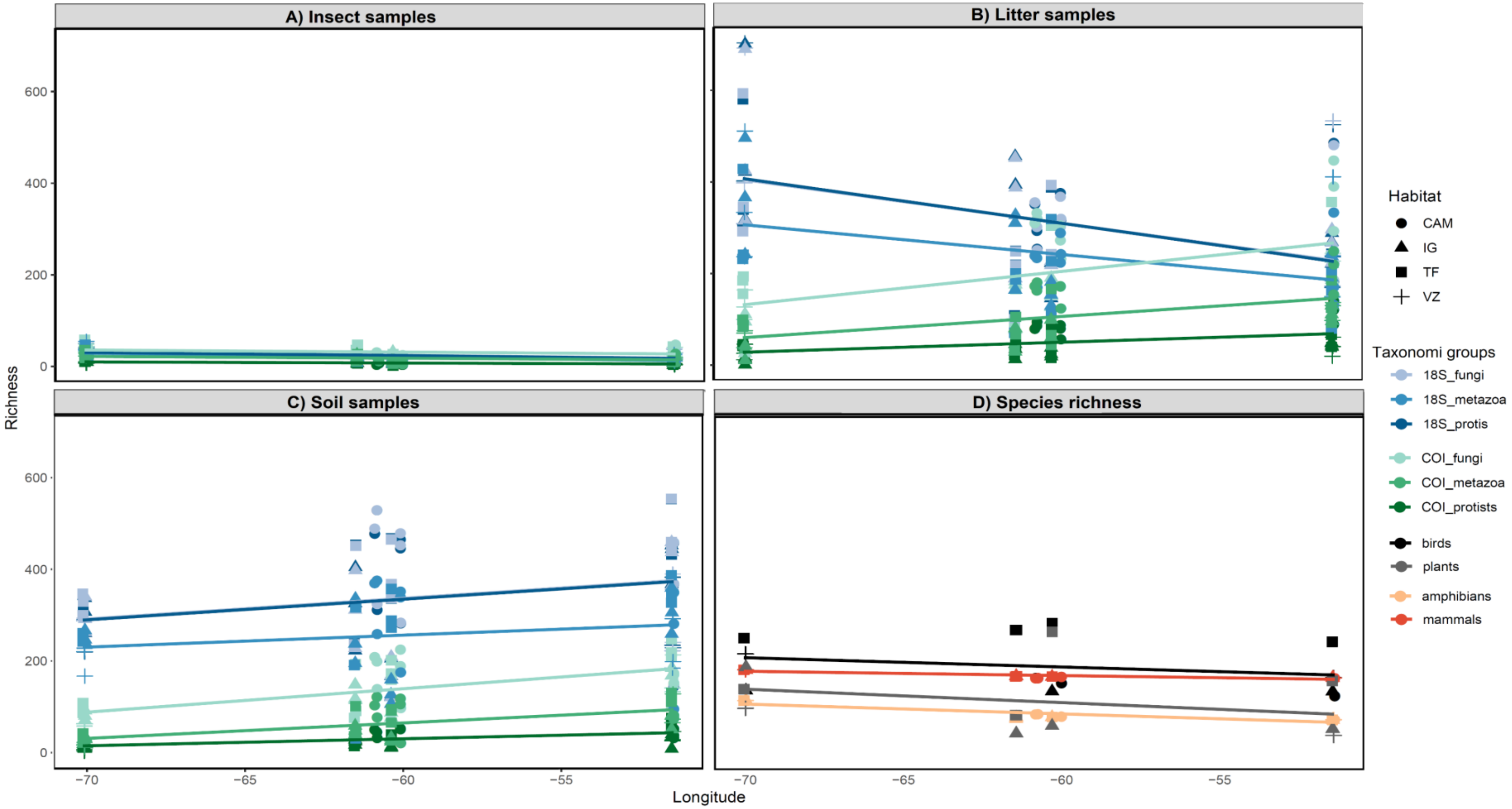

Figure S1 – Metabarcoding OTUs by taxonomic group and species richness of birds and trees per longitude and habitat type. The plots show OTUs richness measured from metabarcoding samples of A) insects, B) litter and C) soil. Plot D) shows the known species richness for

trees and birds from which those samples were obtained and amphibians and mammals from Jenkins et al. (2015). The colour-coding in indicates the taxonomic group and the symbols indicate habitat type (CAM: campinas, IG: igapó, TF: terra firme and VZ: várzea). The results for A-C indicate that OTU richness varies significantly with location and habitat type, and marker. For species richness of trees and birds, a consistency between environment richness (TF > VZ > IG > CAM) can be observed, and a west-to-east gradient, even with a bigger variation, as generally expected based on large-scale inventories. For OTUs an overall pattern with Campinas richer could be observed. The west-to-east gradient is observed in for insect samples and litter for 18S data. The opposite pattern is observed foe COI litter data and soil samples.

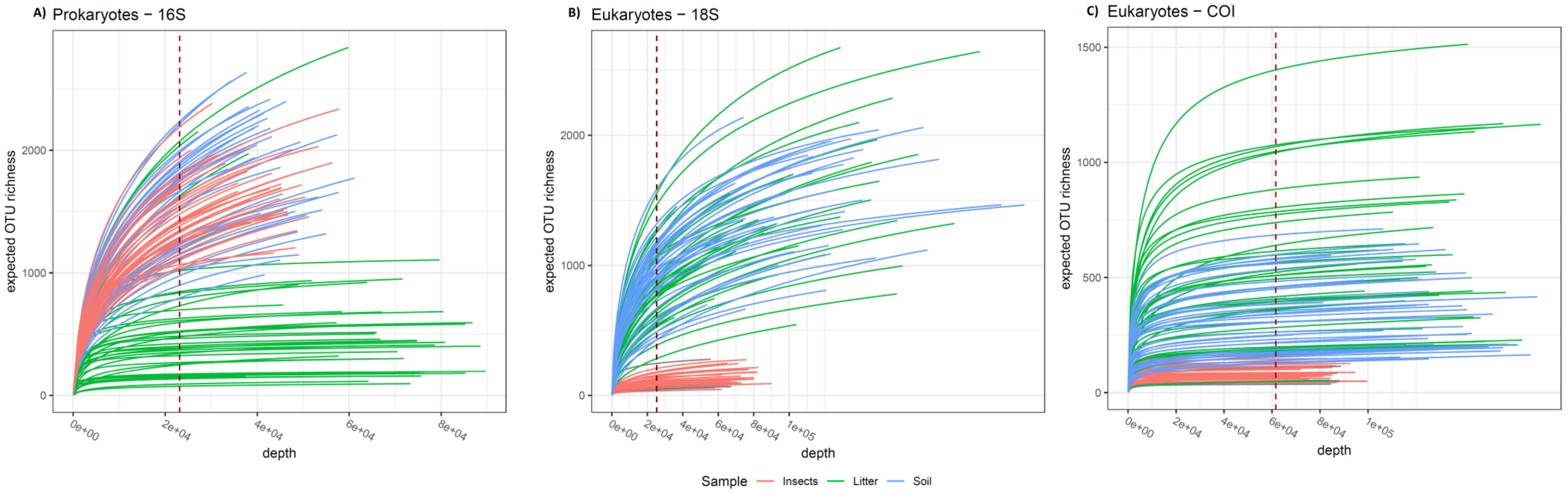

Figure S2. Rarefaction curves. Rarefaction by sample for A) prokaryotes (16S) B) eukaryotes (18S) and C) eukaryotes (COI). The red dashed line shows the minimum number of reads. The 18S marker data are more variable in read number than are the16S and COI data. In the COI data, all plots get close to the asymptote. The different colours represent the different sample type: red are insect samples, green are litter samples and blue are the soil samples.
